# Trim15 stabilizes VDAC3 via ubiquitination to suppress autophagy and enhance chemosensitivity in hypopharyngeal squamous cell carcinoma

**DOI:** 10.1038/s41420-026-02943-0

**Published:** 2026-01-30

**Authors:** Guangyi Wang, Yibang Shen, Lin Wang, Tao Fu, Yichuan Huang, Fangyu Chai, Mingjin Xu, Yan Jiang, Jisheng Zhang

**Affiliations:** 1 Key Laboratory, Department of Otolaryngology-Head and Neck Surgery, Shandong Engineering Research Center for Precision Diagnosis and Treatment in Otolaryngology, the Affiliated Hospital of Qingdao University, Qingdao University, Qingdao, China; 2 Harbin Medical University, Harbin, Heilongjiang China; 3 Weihai Central Hospital, Weihai, Shandong China; 4 Department of Radiation Oncology, the Affiliated Hospital of Qingdao University, Qingdao University, Qingdao, China

**Keywords:** Autophagy, Head and neck cancer

## Abstract

Hypopharyngeal squamous cell carcinoma (HPSCC) is a highly aggressive malignancy with a poor prognosis. This study elucidates the role of the E3 ubiquitin ligase Tripartite Motif Containing 15 (Trim15) and its substrate, mitochondrial voltage-dependent anion channel 3 (VDAC3), in regulating autophagy, mitophagy, and chemoresistance in HPSCC. We found that Trim15 is significantly downregulated in HPSCC tissues and inhibits cell proliferation and migration in FaDu and Detroit 562 cells. Trim15 stabilizes VDAC3 via K6-linked ubiquitination, thereby suppressing autophagy and mitophagy while elevating reactive oxygen species (ROS) levels. VDAC3 knockdown enhances autophagy and mitophagy, concomitantly reducing ROS and promoting cancer cell survival. High-concentration ethanol suppresses Trim15 and VDAC3 expression, suggesting an adaptive response to oxidative stress. Notably, chloroquine (CQ), an autophagy inhibitor, enhances HPSCC sensitivity to 5-fluorouracil (5-FU), with synergistic effects observed in xenograft models. These findings establish the Trim15-VDAC3-mitophagy axis as a critical regulator of HPSCC progression and chemoresistance, offering a novel therapeutic target for augmenting the efficacy of autophagy inhibitors in combination with standard chemotherapy.

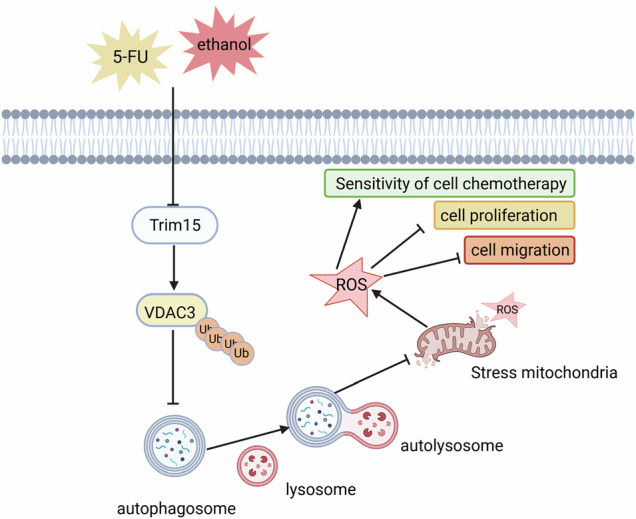

## Introduction

Hypopharyngeal squamous cell carcinoma (HPSCC) is an aggressive head and neck malignancy with a poor prognosis, characterized by a 5-year overall survival rate of 25–40% [1]. Its anatomical location in the hypopharynx often delays symptom onset, leading to advanced-stage diagnosis in most patients [2]. Treatment strategies for advanced HPSCC remain debated, as meta-analyses reveal no significant survival benefit for surgical versus non-surgical approaches, such as radiotherapy and chemotherapy with organ preservation [3]. Non-surgical treatments, however, preserve physiological function, significantly enhancing patients’ quality of life [4]. Given these challenges, elucidating molecular mechanisms underlying HPSCC progression and identifying novel therapeutic targets are imperative for improving clinical outcomes.

Tripartite motif (TRIM) family proteins, characterized by their RING domain, B-boxes, and coiled-coil region, are a diverse group of E3 ubiquitin ligases with over 80 members in humans, playing pivotal roles in cellular processes [5, 6] These proteins are involved in intracellular signaling, development, apoptosis, protein quality control, innate immunity, and autophagy, with their dysregulation linked to various diseases, including cancer [7]. In cancer, TRIM proteins exhibit dual roles, acting as oncogenes or tumor suppressors, influencing tumor initiation and progression [5]. In addition, TRIM proteins modulate cancer therapy responses by targeting key signaling pathways, including p53, NF-κB, and AKT, via ubiquitination [8]. TRIM4 enhances tamoxifen efficacy in breast cancer through K48-linked ubiquitination of SET [9], whereas TRIM65 and TRIM17 confer cisplatin resistance in lung cancer by targeting p53 and RBM38, respectively [10, 11].

Among the TRIM family, Trim15 has emerged as a protein of particular interest in HPSCC due to its unique expression patterns and functional implications. Trim15, a member of the TRIM family, exhibits tumor-type-specific expression patterns. Trim15 functions as an E3 ubiquitin ligase that influences tumor cell behavior in a cancer-specific manner. In non-small cell lung cancer, Trim15 is overexpressed and promotes proliferation and metastasis by ubiquitinating Keap1, which stabilizes Nrf2 and enhances the antioxidant response [12]. In pancreatic tumors, increased Trim15 levels are linked to invasion and metastasis through the ubiquitination of APOA1 and modulation of the IGF2BP2–TLR4 axis, affecting lipid metabolism and mRNA stability [13, 14]. In contrast, studies in colon cancer describe lower Trim15 expression, with Trim15 acting to suppress cell migration or invasion [15].

This study reveals significant Trim15 downregulation in HPSCC, inhibiting FaDu cell proliferation and migration. Trim15 stabilizes VDAC3 via K6-linked ubiquitination, regulating mitophagy and ROS homeostasis. VDAC3 knockdown enhances mitophagy, reduces ROS, and promotes cancer cell survival. High-concentration ethanol suppresses Trim15 and VDAC3 expression, indicating an adaptive response to oxidative stress. Furthermore, CQ enhances HPSCC sensitivity to 5-FU, highlighting the Trim15-VDAC3-mitophagy axis as a key regulator of HPSCC progression and chemoresistance, providing a molecular foundation for targeted therapies.

## Results

### Trim15 is downregulated in HPSCC and inhibits tumor cell proliferation and migration

**Fig. 1:**
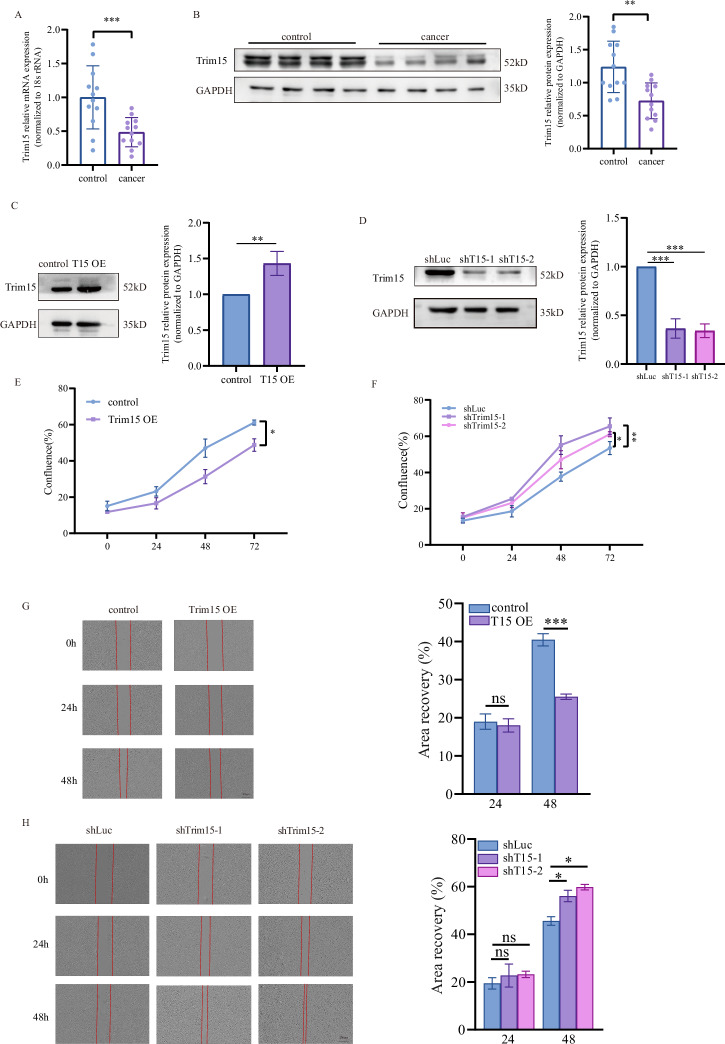
Trim15 is downregulated in HPSCC and suppresses FaDu cell proliferation and migration. **A** Quantitative real-time PCR analysis of Trim15 mRNA in paired HPSCC tumor and adjacent normal tissues (*n* = 12). Data are mean ± SEM; ***P < 0.001, two-tailed Student’s *t* test. **B** Representative Western blot and quantification of Trim15 protein in the same tissue pairs (*n* = 12). Data are mean ± SEM; **P < 0.01, two-tailed Student’s *t* test. Western blot validation and quantification of Trim15 overexpression (T15 OE) (**C**) or shRNA knockdown (**D**) in FaDu cells. Proliferation of FaDu cells with Trim15 overexpression (**E**) or knockdown (**F**) versus controls, measured by IncuCyte live-cell imaging. Data are mean ± SEM (*n* = 3 independent experiments); *P < 0.05, **P < 0.01, two-way ANOVA with Tukey’s test. Wound-healing assays showing migration of FaDu cells with Trim15 overexpression (**G**) or knockdown (**H**). Wound closure quantified at 24 and 48 h. Data are mean ± SEM (*n* = 3); *P < 0.05, ***P < 0.001, Student’s *t* test.

To investigate the role of Trim15 in HPSCC, we quantified Trim15 mRNA and protein expression in paired tumor and adjacent normal tissues from patients with HPSCC using quantitative real-time PCR and western blot analysis. Our results revealed significantly reduced Trim15 mRNA and protein levels in tumor tissues compared to adjacent normal tissues (Figs. 1A, B and S1A). To explore functional implications, we generated FaDu and Detroit 562 cell lines with stable Trim15 overexpression or knockdown using lentiviral vectors. Western blot analysis confirmed successful overexpression and efficient knockdown of Trim15 (Figs. 1C, D and S1B, C). We assessed the impact of Trim15 on tumor cell behavior using live-cell imaging (IncuCyte) to monitor proliferation and wound-healing (scratch) assays to evaluate migration. Trim15 overexpression significantly suppressed cell proliferation compared to control cells (Figs. 1E, and S1D), whereas Trim15 knockdown enhanced proliferation (Figs. 1F and S1E). Similarly, Trim15 overexpression markedly reduced cell migration, while knockdown promoted migration (Figs. 1G, H, and S1F, G). These data collectively indicate that Trim15 is downregulated in HPSCC and exerts tumor-suppressive effects by inhibiting proliferation and migration.

### Trim15 stabilizes mitochondrial channel protein VDAC3 via K6-Linked ubiquitination

**Fig. 2:**
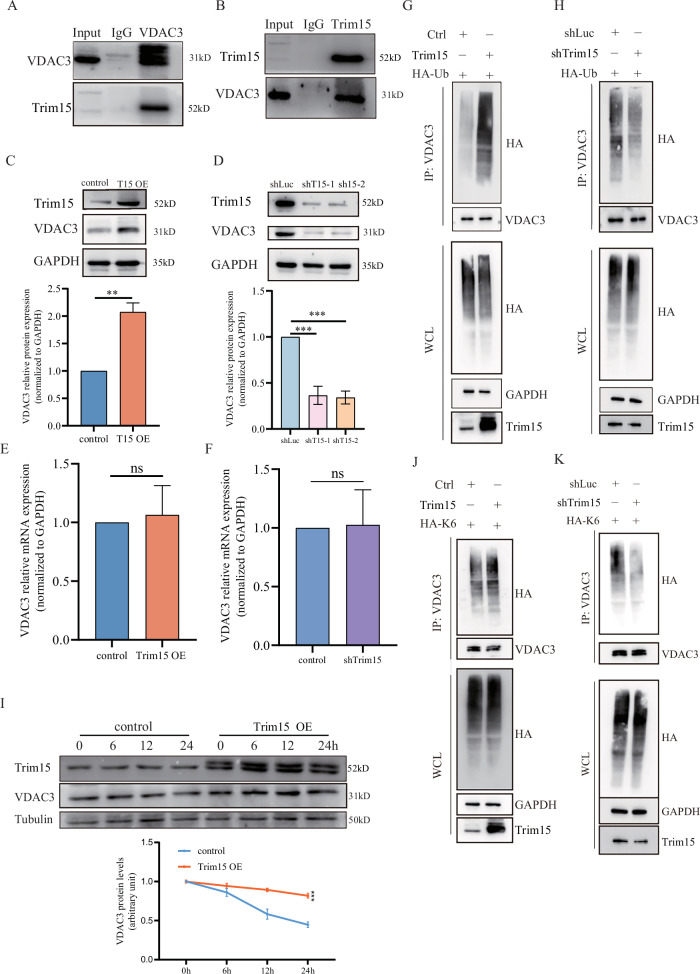
Trim15 stabilizes VDAC3 via K6-linked ubiquitination in FaDu cells. **A** Co-immunoprecipitation assays confirming Trim15-VDAC3 interaction in FaDu cells, with reciprocal IPs in (**B**). Western blot and quantification of VDAC3 protein levels in FaDu cells with Trim15 overexpression (**C**) or knockdown (**D**). Quantitative real-time PCR of VDAC3 mRNA in FaDu cells with Trim15 overexpression (**E**) or knockdown (**F**). Data are mean ± SEM (*n* = 3); n.s. not significant, Student’s *t* test. Ubiquitination assays showing total VDAC3 ubiquitination in FaDu cells with Trim15 overexpression (**G**) or knockdown (**H**). **I** Cycloheximide chase assay demonstrating VDAC3 protein stability in Trim15-overexpressing FaDu cells. The quantification of VDAC3 levels relative to Tubulin levels was determined by ImageJ. Ubiquitination assays detecting K6-linked VDAC3 ubiquitination in FaDu cells with Trim15 overexpression (**J**) or knockdown (**K**).

To understand the molecular basis of Trim15’s tumor-suppressive function, we investigated its interacting partners and downstream effects. We performed co-immunoprecipitation (co-IP) in FaDu cells to identify Trim15-interacting proteins (Fig. S2A). Mass spectrometry analysis revealed a potential interaction between Trim15 and the mitochondrial voltage-dependent anion channel protein 3 (VDAC3). This interaction was validated by reciprocal co-IP using antibodies against Trim15 and VDAC3 (Fig. 2A, B). Further analysis of Trim15 overexpression and knockdown cells revealed that Trim15 positively regulates VDAC3 protein expression (Fig. 2C, D) without significantly affecting its transcription levels (Fig. 2E, F), suggesting post-transcriptional regulation. To further validate the role of VDAC3, we successfully established Detroit 562 cell lines with stable VDAC3 overexpression or knockdown (Fig. S2B, C). Consistent with Trim15’s effects, VDAC3 overexpression suppressed cell proliferation and migration, whereas its knockdown promoted these cellular processes (Fig. S2D–G). As an E3 ubiquitin ligase, we hypothesized that Trim15 modulates VDAC3 stability through ubiquitination. Ubiquitination assays showed that Trim15 overexpression and knockdown increased and decreased total VDAC3 ubiquitination levels, respectively (Fig. 2G, H). However, Cycloheximide (CHX) chase experiments demonstrated that Trim15 overexpression significantly delayed VDAC3 degradation (Fig. 2I), suggesting Trim15 plays a protective role by stabilizing VDAC3 rather than promoting its degradation. Ubiquitin modifications can mediate diverse functions depending on the linkage type. While K48-linked polyubiquitination typically targets proteins for proteasomal degradation, K63-linked chains often regulate signaling pathways, and K6-linked ubiquitination has been associated with protein stabilization in specific contexts. Ubiquitination assays revealed that neither Trim15 overexpression nor knockdown significantly affected the K48- or K63-linked ubiquitination of VDAC3 (Fig. S2I–K). Given our prior finding of K6-linked ubiquitination stabilizing keratin K10 in FaDu cells [16], we investigated whether Trim15 employs a similar mechanism for VDAC3. Ubiquitination assay revealed that Trim15 overexpression increased K6-linked ubiquitination of VDAC3, while knockdown reduced K6-linked ubiquitination (Fig. 2J, K). These results demonstrate that Trim15 stabilizes VDAC3 via K6-linked ubiquitination, contributing to its tumor-suppressive function in HPSCC.

### VDAC3 downregulation promotes cell autophagy

**Fig. 3:**
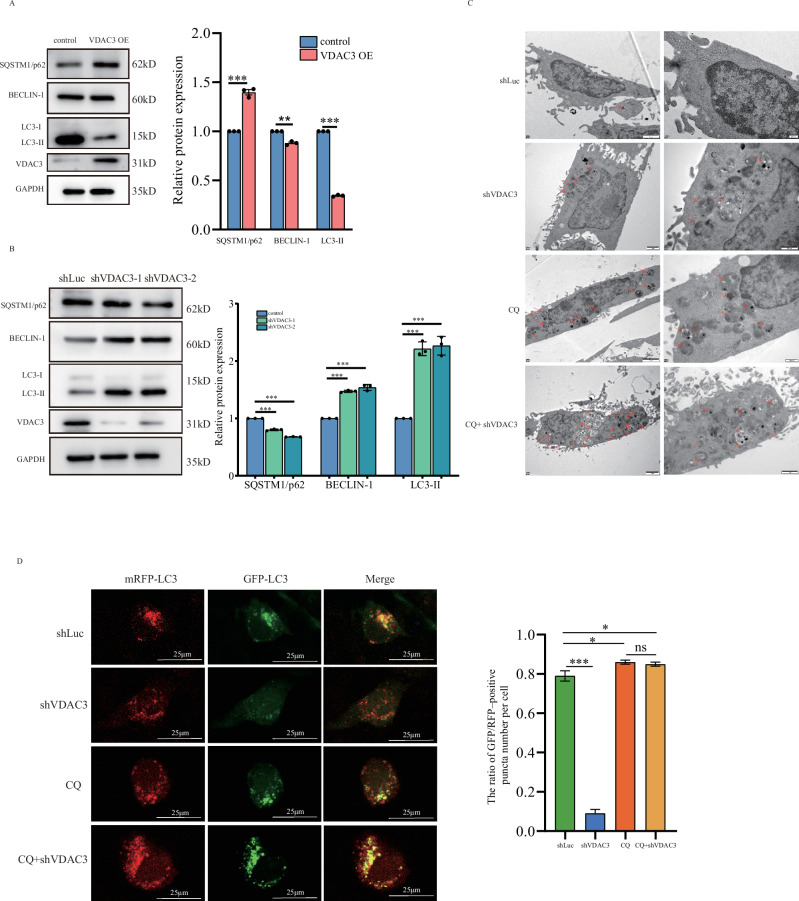
VDAC3 regulates autophagy in FaDu cells. Western blot and quantification analysis of autophagy markers LC3B, Beclin-1, and SQSTM1/p62 in FaDu cells with VDAC3 overexpression (**A**) or knockdown (**B**). **C** Transmission electron microscopy (TEM) images of autophagosomes (indicated by red arrows) in FaDu cells under control (shLuc), VDAC3 knockdown (shVDAC3), chloroquine (CQ), or combined conditions. Scale bars, 500 nm. **D** mRFP-GFP-LC3 assay assessing autophagy flux in conditions as in (**C**). Images and autophagic puncta quantification shown. Data are mean ± SEM; *P < 0.05, ***P < 0.001, Student’s *t* test.

Given VDAC3’s mitochondrial localization, we hypothesized it modulates autophagy in HPSCC. We manipulated VDAC3 expression in FaDu cells and assessed autophagy markers LC3B, Beclin-1, and SQSTM1/p62 by western blot. VDAC3 overexpression significantly decreased LC3-II and Beclin-1 levels while elevating SQSTM1/p62 expression (Fig. 3A), indicative of autophagy suppression. Conversely, VDAC3 knockdown increased LC3-II and Beclin-1 levels and reduced SQSTM1/p62 accumulation, suggesting autophagy enhancement (Fig. 3B). Furthermore, LC3-II expression was significantly downregulated by VDAC3 overexpression but upregulated by its knockdown in Detroit 562 cells (Fig. S3A, B). To confirm these findings, we visualized autophagosome morphology and quantity using transmission electron microscopy (TEM). VDAC3-knockdown FaDu cells exhibited a marked increase in autophagosome formation compared to controls. Notably, combining VDAC3 knockdown with CQ treatment resulted in greater autophagosome accumulation than CQ alone, suggesting that VDAC3 downregulation enhances CQ-mediated inhibition of autophagy flux (Fig. 3C).

We further evaluated autophagy flux using the mRFP-GFP-LC3 dual-fluorescence assay, which distinguishes autophagosomes (yellow fluorescence; mRFP+GFP + ) from autolysosomes (red fluorescence; mRFP+ only) due to GFP quenching in acidic lysosomal environments. VDAC3-knockdown cells displayed significantly reduced green fluorescence compared to controls, indicating enhanced autophagy flux. The enhanced autophagy flux was reversed by the CQ treatment, reinforcing the role of VDAC3 in autophagy regulation (Figs. 3E and S3C). These findings establish VDAC3 as a negative regulator of autophagy in FaDu and Detroit 562 cells.

### VDAC3 signaling regulates ROS levels to maintain FaDu cell proliferation and migration

**Fig. 4:**
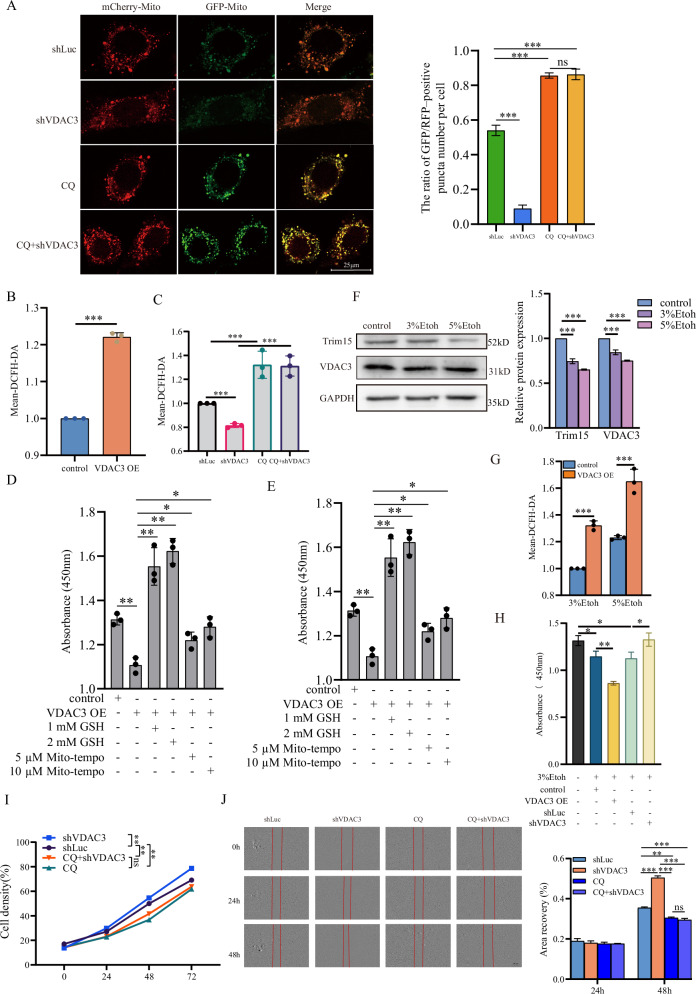
VDAC3 modulates mitophagy, ROS, and cellular behavior in FaDu cells. **A** Cox8-GFP-mCherry assay showing mitophagic flux in FaDu cells under control (shLuc), VDAC3 knockdown (shVDAC3), CQ, or combined conditions. Images and autophagic puncta quantification shown. Data are mean ± SEM; ***P < 0.001, n.s. not significant, Student’s *t* test. Flow cytometry analysis of ROS levels in FaDu cells with VDAC3 overexpression (**B**) or conditions as in (**A**) (**C**). Data are mean ± SEM; ***P < 0.001, Student’s *t* test. Proliferation (**D**) and migration (**E**) assays in VDAC3-overexpressing FaDu cells treated with GSH or Mito-tempo. Data are mean ± SEM (*n* = 3); *P < 0.05, **P < 0.01, Student’s *t* test. **F** Western blot and quantification of Trim15 and VDAC3 in FaDu cells post-alcohol (Etoh) treatment for 24 h. **G** Flow cytometry analysis of ROS levels in FaDu cells with VDAC3 overexpression. Data are mean ± SEM; ***P < 0.001, Student’s *t* test. **H** Cell viability in FaDu cells with VDAC3 overexpression and knockdown under alcohol (3% Etoh) condition. Data are mean ± SEM (*n* = 3); *P < 0.05, **P < 0.01, Student’s *t* test. **I** Proliferation of FaDu cells with control (shLuc), VDAC3 knockdown (shVDAC3), CQ, or combined treatment, measured by IncuCyte live-cell imaging. Data are mean ± SEM; **P < 0.01, n.s. not significant, two-way ANOVA with Tukey’s test. **J** Wound-healing assays showing migration of FaDu cells in conditions as in (**I**) Wound closure quantified at 24 and 48 h. Data are mean ± SEM; ***P < 0.001, **P < 0.01, n.s. not significant, Student’s *t* test.

Given the important role of VDAC1 and VDAC2 in mitophagy, we hypothesized that VDAC3 may also regulate mitophagy, a selective form of autophagy targeting mitochondria [17]. In FaDu and Detroit 562 cells, we employed the Cox8-GFP-mCherry dual-fluorescence assay to monitor mitophagic flux, where reduced green fluorescence indicates mitochondrial delivery to acidic lysosomes. VDAC3-knockdown cells exhibited significantly decreased green fluorescence compared to controls, which was blocked by CQ treatment, consistent with enhanced mitophagic flux (Figs. 4A and S4A).

Our results demonstrate that VDAC3 inhibits mitophagy in FaDu cells. Mitochondrial dysfunction affects intracellular ROS levels, while mitophagy regulates intracellular ROS homeostasis by eliminating abnormal, stressed mitochondria. We examined the effects of VDAC3 on intracellular ROS using flow cytometry with dichlorofluorescein diacetate (DCFH-DA). Results showed that VDAC3 overexpression increased intracellular ROS in FaDu cells (Fig. 4B). Conversely, VDAC3 knockdown decreased intracellular ROS levels, which were restored by CQ treatment (Fig. 4C), suggesting VDAC3 affects intracellular ROS in FaDu cells through autophagy regulation. Tumor cells commonly exhibit redox dysregulation, wherein moderate ROS levels drive oncogenic processes, whereas supraphysiological ROS triggers multiple forms of programmed cell death. We speculated that downregulation of the Trim15-VDAC3 axis is a strategy for cancer cells to avoid excessive ROS levels. Indeed, the ROS scavengers, GSH and Mito-tempo, reversed the VDAC3 overexpression-induced suppression of cell proliferation and migration (Fig. 4D, E).

Alcohol generates elevated reactive oxygen species (ROS) in cancer cells, contributing significantly to HPSCC. Epidemiological studies link chronic alcohol consumption to increased HPSCC risk due to direct exposure of the hypopharyngeal mucosa to ethanol, causing cytotoxicity, oxidative stress, and mitochondrial dysfunction [18–20]. Unlike systemic organs, the hypopharyngeal mucosa is directly exposed to concentrated ethanol during swallowing. To model this high alcohol exposure, we cultured FaDu HPSCC cells in DMEM supplemented with 3% or 5% ethanol. Notably, alcohol treatment significantly reduced Trim15 and VDAC3 levels in both FaDu and Detroit 562 cells (Figs. 4F and S4B. Overexpression of VDAC3 resulted in increased ROS levels in alcohol-treated FaDu and Detroit 562 cells (Figs. 4G and S4C). In the presence of alcohol, VDAC3 overexpression decreased, while VDAC3 knockdown increased, FaDu cell viability (Fig. 4H). Our results suggest that the decrease of the Trim15-VDAC3 axis is an effective system for keeping redox balance to sublethal steady-state conditions. This adaptation may enhance cellular tolerance to alcohol-induced oxidative stress, promoting tumor cell survival and progression.

VDAC3 knockdown-induced autophagy is critical for maintaining cellular homeostasis in FaDu and Detroit 562 cells. To explore whether autophagy inhibition could effectively suppress cell behaviors in these cell lines, we proceeded with experimental testing. Cell proliferation and scratch assays demonstrated that CQ treatment abolished VDAC3 knockdown’s augmentation of cell proliferation and migration, suggesting that VDAC3 may regulate these processes through autophagy. Notably, CQ alone significantly inhibited cell proliferation and migration, highlighting its potential clinical value in hypopharyngeal cancer treatment (Figs. 4I, J and S4D, S4E). These data suggest that the Trim15-VDAC3 axis maintains redox homeostasis, with its downregulation promoting tumor cell survival under oxidative stress.

### VDAC3 enhances HPSCC cell sensitivity to 5-fluorouracil

**Fig. 5:**
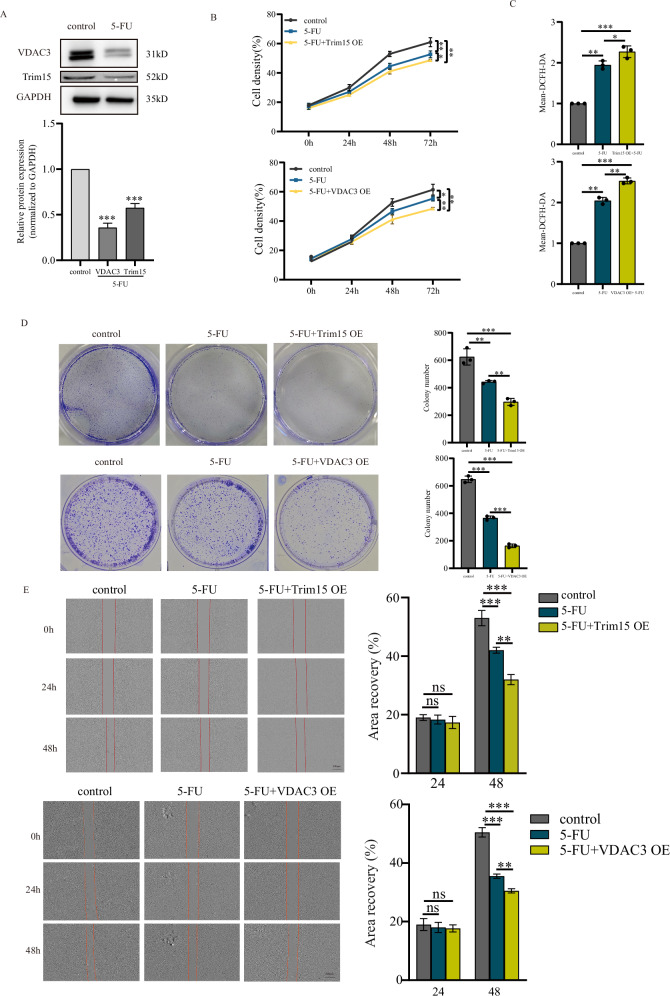
Trim15-VDAC3 axis enhances 5-fluorouracil (5-FU) sensitivity in FaDu cells. **A** Western blot and quantification of Trim15 and VDAC3 in FaDu cells post-5-FU treatment. **B** Proliferation assay in Trim15- or VDAC3-overexpressing FaDu cells with 10 μM 5-FU. Data are mean ± SEM (*n* = 3); *P < 0.05, **P < 0.01, two-way ANOVA with Tukey’s test. **C** Flow cytometry of ROS levels in conditions as in (**B**). Data are mean ± SEM (*n* = 3); *P < 0.05, **P < 0.01, ***P < 0.001, Student’s *t* test. **D** Colony formation assay showing clonogenic survival in Trim15- or VDAC3-overexpressing FaDu cells with 10 μM 5-FU. Data are mean ± SEM (*n* = 3); **P < 0.01, ***P < 0.001, Student’s *t* test. **E** Wound-healing assay of Trim15- or VDAC3-overexpressing FaDu cells with 10 μM 5-FU. Wound closure quantified at 24 and 48 h. Data are mean ± SEM (*n* = 3); ***P < 0.001, **P < 0.01, n.s. not significant, Student’s *t* test.

Our findings demonstrate that VDAC3 suppresses mitophagy and elevates intracellular ROS levels in hypopharyngeal squamous cell carcinoma cells. Given mitophagy’s role in modulating chemosensitivity, we investigated VDAC3’s impact on the response to 5-FU, a standard chemotherapeutic agent for HPSCC. Western blot analysis revealed that 5-FU treatment significantly reduced Trim15 and VDAC3 expression in FaDu cells (Fig. 5A). We hypothesized that 5-FU-induced downregulation of the Trim15-VDAC3 axis may contribute to chemoresistance and that restoring its expression could enhance 5-FU sensitivity.

The results revealed that, compared to 5-FU alone, Trim15 or VDAC3 overexpression combined with 5-FU further inhibited FaDu cell proliferation (Fig. 5B) and increased intracellular ROS levels (Fig. 5C). Colony formation and scratch assays demonstrated that Trim15 or VDAC3 overexpression combined with 5-FU further suppressed FaDu cell colony formation and migration capabilities (Fig. 5D, E). Given the essential function of VDAC3 in mitophagy regulation, we further validated its role in 5-FU response in Detroit 562 cells. VDAC3 expression was downregulated upon 5-FU treatment (Fig. S5A). Consistent with the results in FaDu cells, we observed similar effects on cell proliferation, ROS production, colony formation, and cell migration in Detroit 562 cells (Fig. S5B–E). Collectively, these results indicate that the Trim15-VDAC3 axis enhances 5-FU sensitivity, positioning it as a potential modulator of HPSCC chemotherapy.

### Chloroquine as a potential therapeutic agent in vivo for HPSCC

**Fig. 6:**
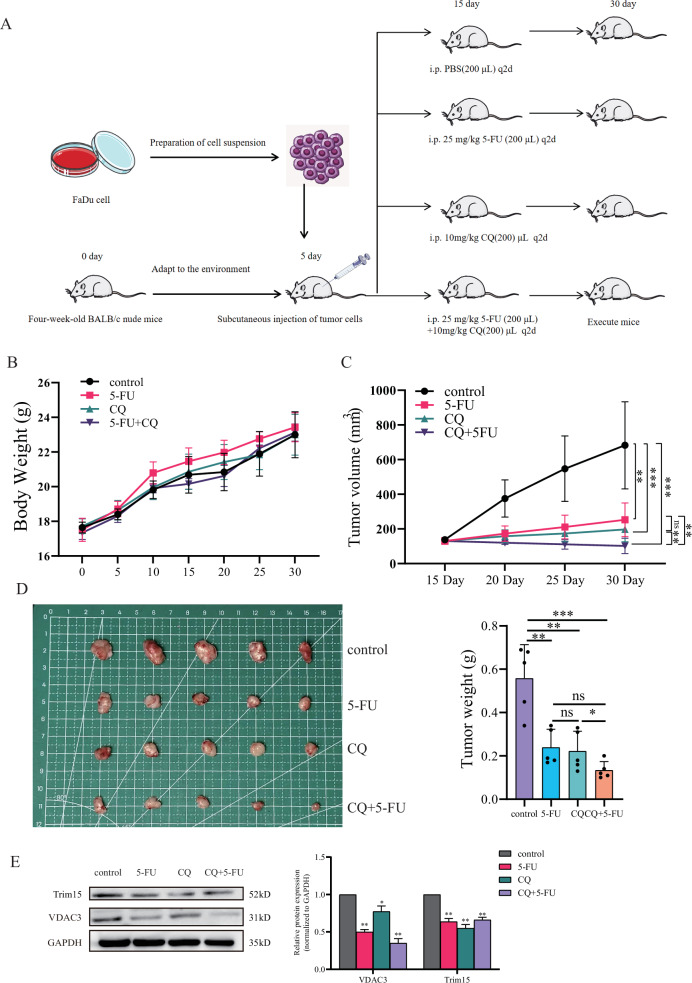
CQ and 5-FU combination therapy inhibits HPSCC tumor growth in vivo. **A** Experimental procedure. **B** Body weight monitoring, indicating no significant toxicity. Data are mean ± SEM (*n* = 5/group). **C** Tumor volume progression under different treatments. Data are mean ± SEM (*n* = 5/group); ***P < 0.001, **P < 0.01, two-way ANOVA with Tukey’s test. **D** Tumor images and weights. Data are mean ± SEM (*n* = 5/group); ***P < 0.001, **P < 0.01, *P < 0.05, n.s. not significant, Student’s *t* test. **E** Western blot and quantification of Trim15 and VDAC3 in xenograft tumors. Data are mean ± SEM (*n* = 3); n.s. not significant, *P < 0.05, **P < 0.01, Student’s *t* test.

Our studies have shown the potential of CQ in treating HPSCC cells. To explore the synergistic potential of CQ with conventional chemotherapy, we evaluated its antitumor efficacy, both as a monotherapy and in combination with 5-FU, in a xenograft model (Fig. 6A). The body weight of mice did not differ significantly across all groups, suggesting that neither CQ nor the combination therapy induced overt systemic toxicity (Fig. 6B). Our results revealed that both 5-FU and CQ monotherapies significantly reduced tumor volume compared to the PBS control group (Fig. 6C). Notably, the combination of 5-FU and CQ elicited a more pronounced tumor regression effect, indicating a synergistic interaction between the two agents. Tumor weight measurements corroborated these findings, with the 5-FU + CQ group exhibiting significantly lower tumor weights than the CQ monotherapy group (Fig. 6D). Importantly, the combination of 5-FU and CQ significantly downregulated Trim15 and VDAC3 expression in vivo (Fig. 6E). These findings demonstrate that CQ exerts robust anti-HPSCC activity and enhances the chemosensitivity of 5-FU in vivo. The synergistic effect of CQ and 5-FU highlights its potential as an adjunctive therapeutic agent for HPSCC.

## Discussion

Our study elucidates the novel role of Trim15, an E3 ubiquitin ligase, in regulating VDAC3 via K6-linked ubiquitination in HPSCC, with significant implications for autophagy, mitophagy, and chemotherapy resistance. The downregulation of Trim15 in HPSCC, contrasting with its upregulation in non-small cell lung and pancreatic cancers, underscores its context-dependent role in tumorigenesis [12, 13]. This variability aligns with observations in gastric adenocarcinoma, where low Trim15 expression correlates with advanced TNM stage and poor survival [21], and in pancreatic ductal adenocarcinoma, where elevated Trim15 promotes metastasis via APOA1 degradation [14]. These findings suggest that Trim15’s oncogenic or tumor-suppressive functions are tumor-type specific, likely influenced by substrate availability and cellular context.

The identification of VDAC3 as a Trim15 substrate, mediated by K6-linked ubiquitination, represents a significant advance in understanding mitochondrial quality control in cancer. Unlike degradative K48-linked ubiquitination, K6-linked chains modulate protein stability, localization, and interactions, as evidenced by their roles in DNA damage responses and mitochondrial processes [22–24]. Our cycloheximide chase assays demonstrate that Trim15 stabilizes VDAC3, potentially enhancing its role as a redox sensor and mitophagy regulator. This is consistent with prior reports of K6-linked ubiquitination stabilizing keratin 10 in HPSCC via TRIM21 and modulating Parkin’s mitochondrial translocation or mitofusin-2’s fusion activity [25, 26]. VDAC1 and VDAC3 are dispensable for mitophagy and mitochondrial clustering [27], although VDAC1, VDAC2, and VDAC3 each support Parkin recruitment and mitophagy [28, 29]. A recent study identifies TRIM31 as an alternative E3 ligase that directs proteasomal degradation of VDAC1 [30], a process distinct from the mitophagy pathway engaged by Parkin. These results suggest that VDAC isoforms play context-dependent roles in Parkin recruitment, with TRIM31 offering a distinct mechanism for VDAC regulation in mitochondrial quality control. The Trim15-VDAC3 axis thus introduces a novel pathway for mitophagy regulation, distinct from the PINK1/Parkin-VDAC pathway, where VDAC1 polyubiquitination drives autophagosome formation [31, 32]. However, the precise mechanisms by which Trim15-VDAC3 regulates mitophagy require further investigation.

VDAC3 plays a structurally defined and context-dependent role in redox regulation, while its participation in mitophagy overlaps with functions performed by VDAC1 and VDAC2. VDAC3’s redox-sensitive cysteine residues position it as a critical mediator of mitochondrial responses to oxidative stress, with implications for cancer cell metabolism and therapy resistance. In yeast, replacing the VDAC3 N-terminal region with that of VDAC1 enhances ROS modulation and extends lifespan [33], whereas overexpression of VDAC3 has been reported to increase sensitivity to oxidative stress [34]. At low ROS levels, oxidized VDAC3 recruits Parkin for proteasomal degradation; at high ROS levels, it integrates into lysosome-targeted mitochondria-derived vesicles; and at extreme ROS thresholds, extensive oxidation triggers full mitophagy [35, 36]. Our findings that VDAC3 inhibition enhances autophagy and mitophagy, promoting HPSCC cell proliferation and migration, suggest that Trim15-mediated stabilization of VDAC3 may counteract excessive mitophagy, thereby limiting cancer cell survival under stress. Conversely, 5-FU and ethanol reduce VDAC3 expression, likely as an adaptive strategy to enhance mitophagy and mitigate oxidative stress. These differential responses highlight VDAC3’s role as a dynamic regulator of mitochondrial quality control in response to therapeutic stressors.

The dual role of autophagy in cancer therapy—protective against apoptosis versus cytotoxic via resource depletion—complicates its therapeutic targeting [31, 37, 38] Our results demonstrate that autophagy inhibitors, such as chloroquine, enhance the efficacy of chemotherapy in HPSCC, possibly by suppressing protective autophagy, consistent with reports in breast cancer [39]. Our research confirmed that chloroquine, alone or in combination with 5-FU, reduces HPSCC cell viability, while VDAC3 overexpression synergizes with these treatments, likely by modulating mitophagy and oxidative stress responses. These findings align with the role of TRIM11 in nasopharyngeal carcinoma, where p62-mediated autophagy promotes radiotherapy resistance via the β-catenin/ABCC9 axis [40]. The Trim15-VDAC3 axis may similarly regulate autophagy-dependent therapy resistance, offering a targetable node for therapeutic intervention.

Given these mechanistic insights, targeting the Trim15-VDAC3 axis holds promise for therapeutic intervention in HPSCC. The therapeutic potential of targeting Trim15-VDAC3 interactions is underscored by its modulation of mitophagy and cancer cell metabolism. VDAC3’s closure promotes the Warburg effect, enhancing cancer cell survival under hypoxic conditions, while its opening favors oxidative phosphorylation, potentially sensitizing cells to therapy [41, 42]. By stabilizing VDAC3, Trim15 may shift this balance, influencing HPSCC’s metabolic phenotype and therapy response. Future studies should employ multi-omics approaches to map the Trim15-VDAC3 interactome and its downstream effects on mitochondrial dynamics. Dynamic imaging techniques, such as live-cell mitophagy assays, could clarify the spatiotemporal regulation of VDAC3 ubiquitination. Additionally, the mechanistic details of Trim15’s role in K6-linked ubiquitination, including its interplay with deubiquitinases like USP8, warrant further investigation [25]

Limitations of our study include the need for in vivo validation of the Trim15-VDAC3 axis and its therapeutic targeting. Clinical studies evaluating Trim15 expression and VDAC3 ubiquitination in HPSCC patient cohorts could establish their prognostic value. Moreover, the development of small-molecule inhibitors or activators of Trim15 could enable precise modulation of VDAC3 function, enhancing the efficacy of existing therapies. Integrating these approaches with multi-omics and real-time monitoring technologies will unravel the complex autophagy regulatory network, paving the way for personalized treatment strategies in HPSCC and other cancers.

In conclusion, the Trim15-VDAC3 axis represents a novel regulator of mitophagy and therapy resistance in HPSCC, with K6-linked ubiquitination stabilizing VDAC3 to modulate mitochondrial quality control. By targeting this pathway, particularly in combination with autophagy inhibitors like chloroquine, we may overcome resistance to chemotherapy, offering new avenues for clinical translation.

## Materials and methods

### Clinical samples

Tumor and matched adjacent normal tissues (>2 cm from tumor margins) were collected from 12 newly diagnosed HPSCC patients at the Department of Otolaryngology, The Affiliated Hospital of Qingdao University. Patients had no prior radiotherapy or chemotherapy. Histological diagnoses were independently confirmed by two senior pathologists. The study was approved by the Medical Ethics Committee of the Affiliated Hospital of Qingdao University, with informed consent obtained from all participants.

### Cell culture and transfection

The human pharyngeal squamous cell carcinoma FaDu (RRID: CVCL_1218) and Detroit 562 (RRID: CVCL_1171) cell lines were obtained from Shanghai Cell Bank. Cells were cultured in DMEM medium (GIBCO, C11995500BT) containing 10% fetal bovine serum (ExCell Bio FSP500) and maintained at 37 °C with 5% CO₂. Short tandem repeat (STR) typing and cell line authentication were completed within three years.

For gain-of-function studies, human VDAC3 and Trim15 cDNAs were cloned into pLenti-CAG-Puro vector. For loss-of-function experiments, two independent shRNA sequences targeting VDAC3 or Trim15 were subcloned into pLKO.1 with AgeI/EcoRI digestion. Lucifersase-targeting shRNA served as control. The constructs were verified by Sanger sequencing and packaged into lentivirus using 293 T cells with psPAX2 and pMD2.G. FaDu cells were infected with lentivirus and the pure cell population was obtained with 2 μg/mL puromycin selection for 72 h. The shRNA constructs targeting sequences as follows: shTRIM15-1: 5′-GCTTCTACAAGATGTCAGA-3′, shTRIM15-2: 5′-GGATGTAAAGTGTCAAGAA-3′, shVDAC3-1‌: 5′-GCAACCTAGAAACCAAATATA-3′, shVDAC3-2‌: 5′-CCAGAAGGTGAATGAGAAGAT-3′, shLuciferase(Luc): 5′-CTTACGCTGAGTACTTCGA-3′.

### RNA extraction and real-time PCR

RNA was extracted from tissues using TRIzol (Vazyme, Nanjing, China) following manufacturer’s instructions. One microgram of RNA was reverse-transcribed using HiScript II Q RT SuperMix (R223, Vazyme). Real-time PCR reactions utilized ChamQ SYBR qPCR Master Mix (Q711, Vazyme). GAPDH for cells and 18 s rRNA for tissues served as the internal reference gene. Relative gene expression was calculated using the 2^(-ΔΔCT) formula. The qPCR primers were designed as follows: Trim15 forward 5’-GCAGCCAGCAAGTGAGCTT-3’ and reverse 5’-TGATGCGCCAAGTTTTCTGAG-3’, 18 s rRNA forward 5’-CTGGATAACCAGCAGCTAGGAA-3’ and reverse 5’-GAATTTCACCTCTAGCGGCG-3’, GAPDH forward 5’-TCGACAGTCAGCCGCATCTT-3’ and reverse 5’-GAGTTAAAAGCAGCCCTGGTG-3’, and VDAC3 forward 5’-GCTTGGACAGCTGGGAGTAA-3’ and reverse 5’-AGTCCAATCAGGCTGGCATT-3’.

### Western blot

Total protein was extracted using lysis buffer containing 2% SDS, with protein concentration determined by the Lowry method (PC0030, Solarbio). Equal protein amounts were separated by Tris-Tricine SDS-PAGE and transferred to PVDF membranes. Membranes were blocked with 5% skim milk for 1 h at room temperature, then incubated overnight at 4 °C with the following antibodies: GAPDH (AF0343, Elabscience), α-tubulin (01270, CWBio), Trim15 (13623-1-AP, ProteinTech), VDAC3 (823531, Zenbio), SQSTM1 (380612, Zenbio), LC3B (R381544, Zenbio) and Beclin 1 (R381896, Zenbio). After TBST washing, membranes were incubated with corresponding secondary antibodies for 1 h at room temperature. Target proteins were detected using ECL hypersensitive chemiluminescence kit (E422, Vazyme). ImageJ software was used for band densitometric analysis.

### Cell proliferation analysis

Live cell proliferation experiments were conducted using a live cell workstation. Cells were seeded in 96-well plates at 5000 cells/well and treated accordingly after adherence. The system maintained 37 °C and 5% CO₂ culture conditions, with images captured every 12 h over 48 h continuous monitoring. Statistical visualization was performed using GraphPad Prism 10.

### Cell migration assay

Cells were grown in 12-well plates until confluence, then subjected to 24-hour starvation. A straight line scratch was created using a pipette tip. After PBS washing, cells were cultured in DMEM containing 0.5% fetal bovine serum. Wound areas were photographed at specified time points and analyzed using ImageJ software.

### ROS detection

Reactive oxygen species (ROS) levels were determined using an ROS detection kit (S0033, Beyotime) containing dichlorofluorescein diacetate (DCFH-DA). After three PBS washes, cells were treated with 10 μM DCFH-DA at 37 °C for 30 min in a cell incubator. Cells were subsequently trypsinized and resuspended in PBS for flow cytometric analysis. A total of 10,000 cells were analyzed with mean fluorescence intensity calculated.

### Immunoprecipitation and immunoblotting

Cells were washed with cold PBS and lysed in RIPA buffer containing protease/phosphatase inhibitors (MCE, HY-K0021). Cell lysates were sonicated and centrifuged at 12,000 rpm at 4 °C for 15 min. Supernatants were collected and incubated overnight at 4 °C with protein A/G magnetic beads (MCE, HY-K0202) bound to commercial antibodies. The next day, beads were washed thrice with PBS and resuspended in 50 μl of 2× SDS-PAGE buffer. Mixtures were boiled at 98 °C for 10 min, centrifuged, and supernatants collected for protein blot or mass spectrometry analysis.

### Transmission electron microscopy

Pretreated cell samples were digested into 15 ml centrifuge tubes, centrifuged for 10 min, supernatants discarded, and pre-fixed with electron microscopy fixative (Solarbio, China) at 4 °C. Samples were rinsed thrice with 0.1 M phosphate buffer (PBS, pH 7.4) for 15 min each. Secondary fixation used 1% osmium tetroxide for 2 h followed by three PBS washes. Samples underwent gradual ethanol dehydration (30%, 50%, 70%, 80%, 90%, 95%, and 100%) for 15 min each. Specimens were infiltrated with epoxy resin, embedded, and polymerized at 60 °C for 48 h. Ultrathin sections (60–80 nm) were cut using an ultramicrotome, stained with uranyl acetate (15 min) and lead citrate (10 min). Cell ultrastructure was observed and photographed using transmission electron microscopy.

### In vivo ubiquitination detection

Cells were treated with 25 μM MG132 for 2 h before collection. Cells were subsequently washed with cold PBS and lysed in ubiquitination lysis buffer containing 20 mM N-ethylmaleimide (HY-D0843, MCE), a deubiquitinating enzyme inhibitor. Lysates were rapidly heated at 98 °C for 10 min, sonicated, and incubated at 4 °C for 1 h. Samples were centrifuged at 12,000 rpm at 4 °C for 30 min. Supernatants were incubated overnight at 4 °C with protein A/G magnetic beads (MCE, HY-K0202) bound to commercial antibodies. The following day, immunoprecipitation products were resuspended in 2× SDS-PAGE buffer and boiled at 98 °C for 10 min for protein blot detection.

### Autophagy flux detection

Cells were infected with mRFP-GFP-LC3 or Cox8-mCherry-GPF dual-fluorescent autophagy adenovirus (Hanbio Technology, 101508AP) for 48 h, then fixed with 4% paraformaldehyde. Images were acquired using a confocal fluorescence microscope and quantified using ImageJ.

### Colony formation assay

FaDu cells were seeded in 6-well plates (2000 cells/well) and incubated at 37 °C for 24 h. Both treatment and control groups were cultured in identical volumes of complete medium for approximately 10 days. Cells were subsequently fixed with general tissue fixative (Wuhan Servicebio Technology Co., Ltd.), stained with 0.1% crystal violet, and photographed.

### Xenograft model

Animal experiments were conducted according to protocols approved by the Animal Experimental Ethics Committee of Qingdao University. Five four-week-old female BALB/c nude mice per group were randomly assigned and subcutaneously injected with 100 μl cell suspensions containing 5 × 10^6^ FaDu cells. Two weeks post-tumor formation, to evaluate the combined effects of chloroquine and 5-fluorouracil, mice were randomly divided into four groups (5 mice per group): PBS control, 5-FU (25 mg/kg q2d), CQ (10 mg/kg), and 5-FU + CQ. During each administration, mouse weight and tumor diameter were recorded. On day 15 post-treatment initiation, mice were euthanized, subcutaneous tumors excised for measurement and weighing, and statistical analyses performed based on recorded data.

### Statistical analysis

Statistical analyses used GraphPad Prism software. Student’s *t* test evaluated significant differences between two data groups, while two-way ANOVA assessed significant differences across multiple groups. All data are expressed as mean ± standard error of the mean (SEM). *P* < 0.05 indicated statistical significance, while *p* > 0.05 was considered non-significant.

## Supplementary information

Supplementary Figure 1

Supplementary Figure 2

Supplementary Figure 3

Supplementary Figure 4

Supplementary Figure 5

Original images of Fig1

Original images of Fig2

Original images of Fig3

Original images of Fig5

Original images of Fig4

Original images of Fig6

Original Image of Supplementary Fig1

Original Image of Supplementary Fig2

Original Image of Supplementary Fig3

Original Image of Supplementary Fig4

Original Image of Supplementary Fig5

Supplementary Figure Legends
