# Supplementary figures and images for: Trim15 stabilizes VDAC3 via ubiquitination to suppress autophagy and enhance chemosensitivity in hypopharyngeal squamous cell carcinoma
Source: Cell Death Discov. 2026 Jan 30;12:88. doi: 10.1038/s41420-026-02943-0 (PMC12876940; doi:10.1038/s41420-026-02943-0)

A

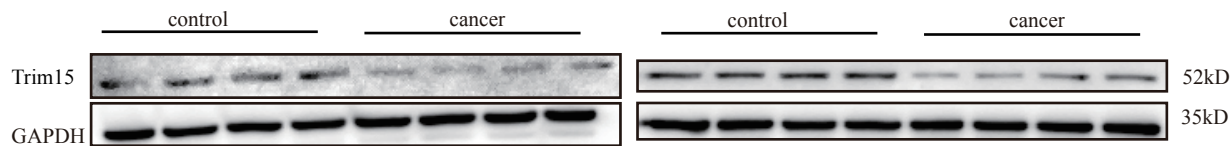

B

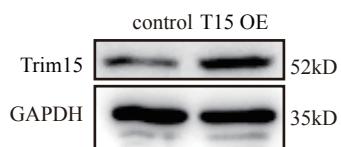

C

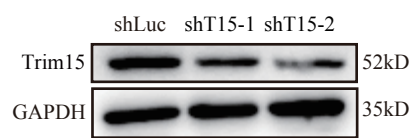

D

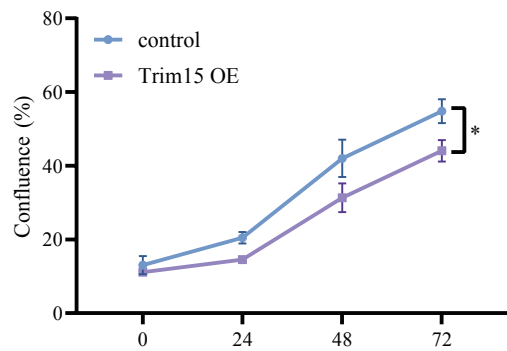

E

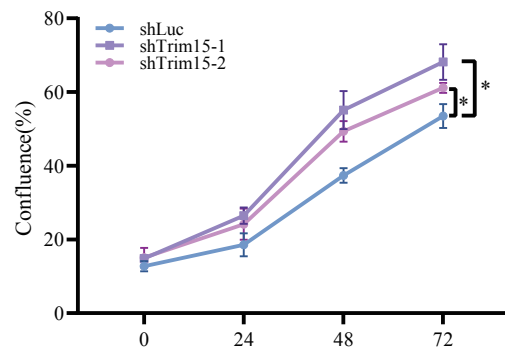

F

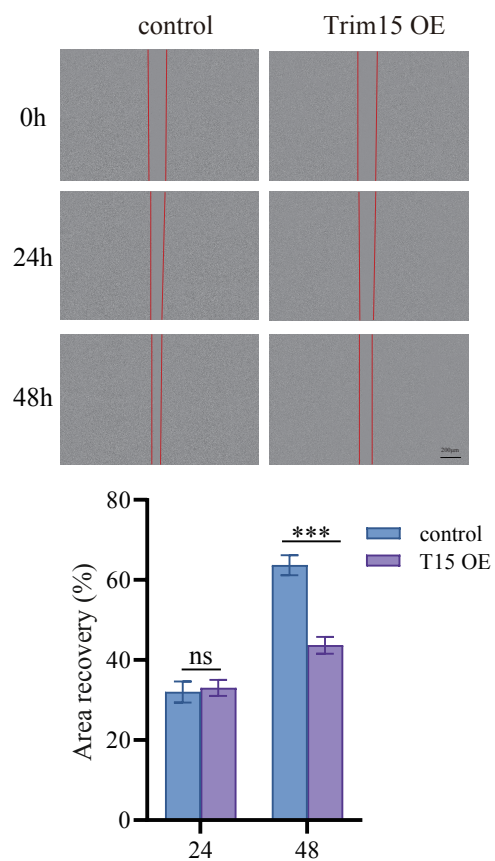

G

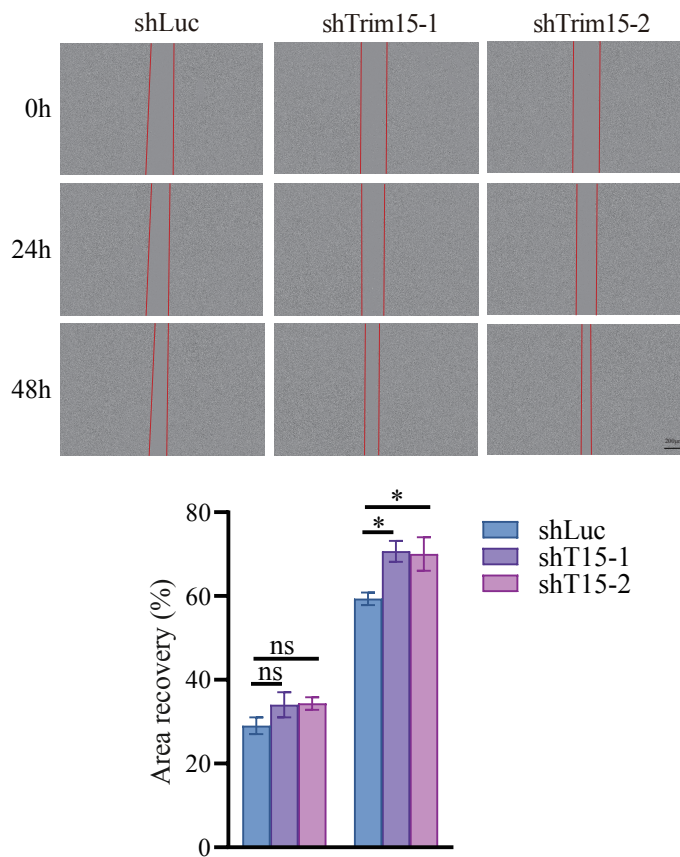

Supplement: Supplementary file 1 — Supplementary Figure 1 [file 41420_2026_2943_MOESM1_ESM.pdf]

A

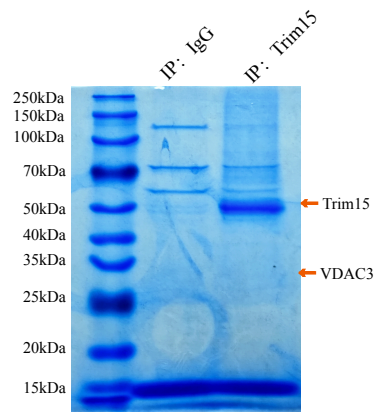

B

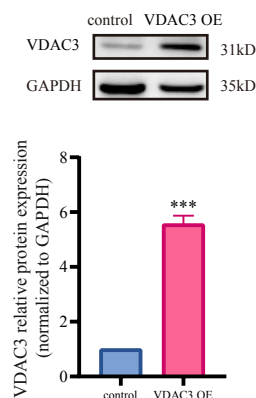

C

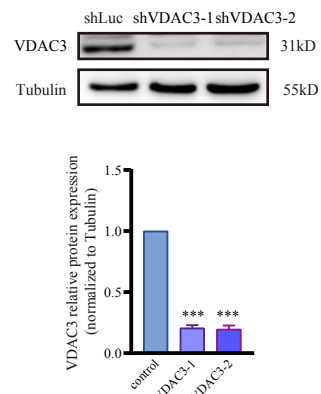

D

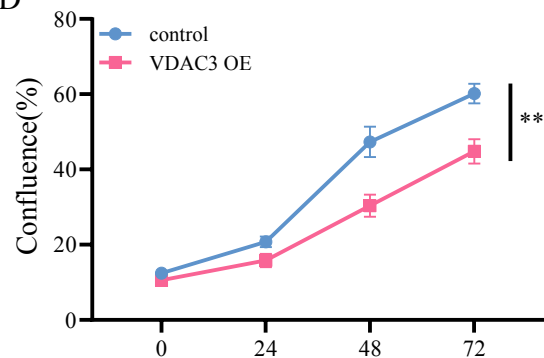

F

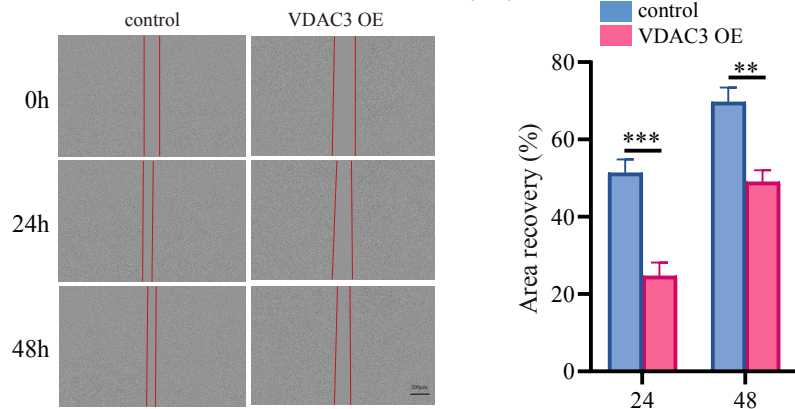

E

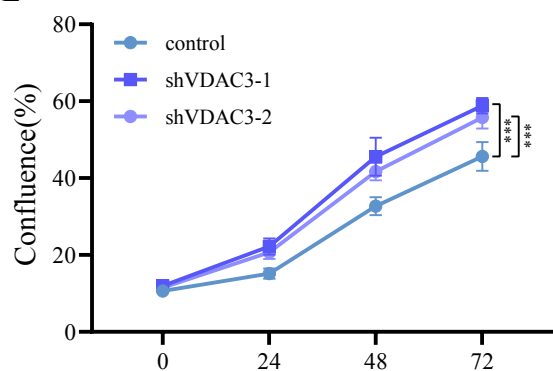

G

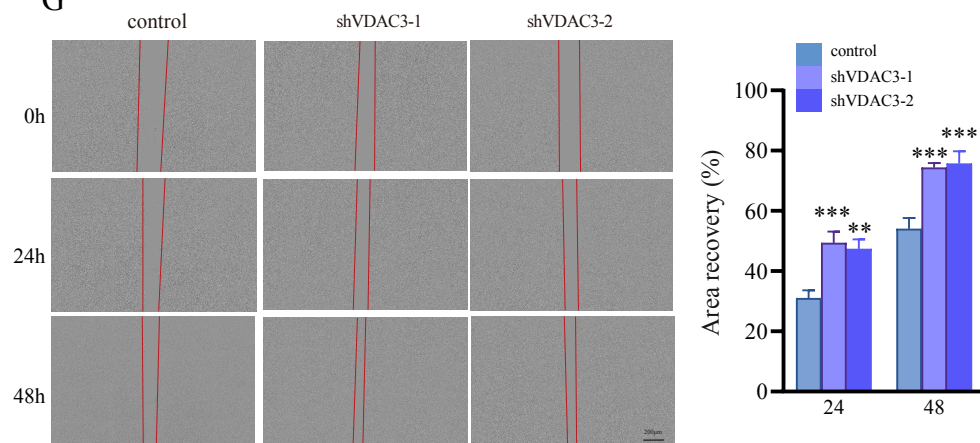

H

I

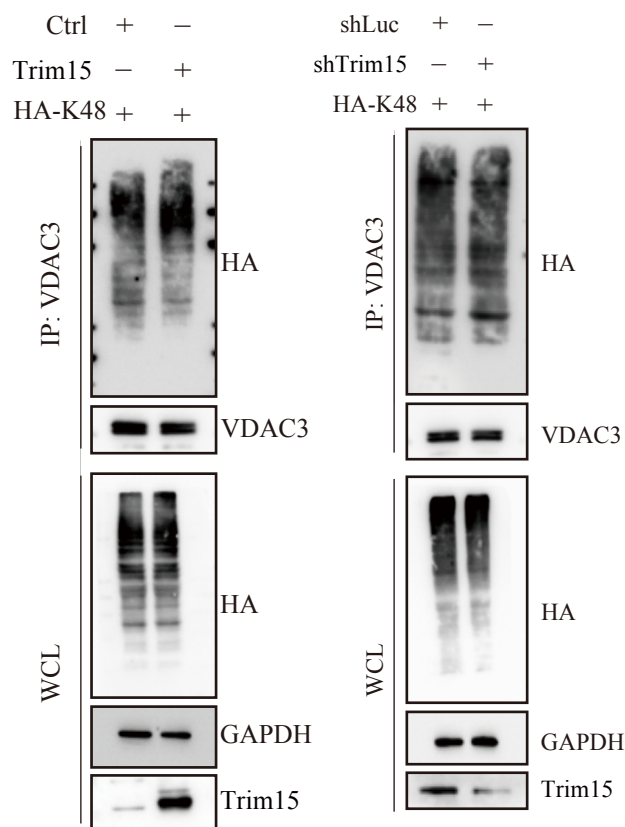

J

Ctrl + -  
Trim15 - +  
HA-K63 + +

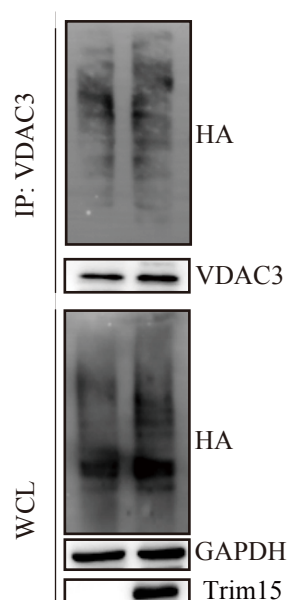

K

shLuc + -  
shTrim15 - +  
HA-K63 + +

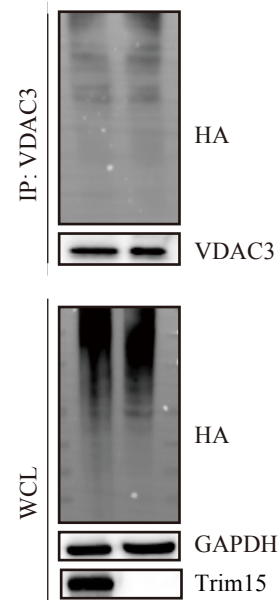

Supplement: Supplementary file 2 — Supplementary Figure 2 [file 41420_2026_2943_MOESM2_ESM.pdf]

A

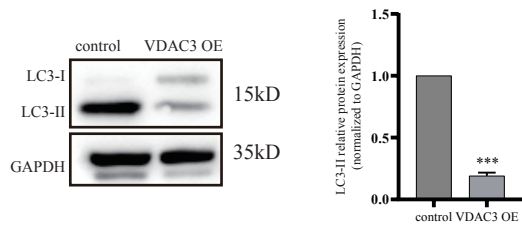

B

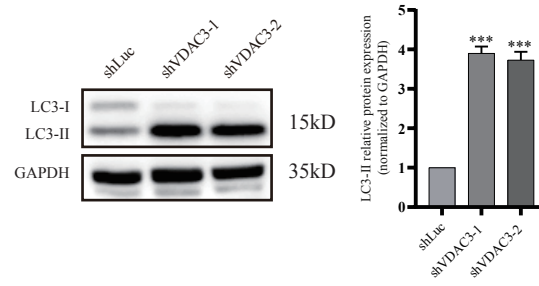

C

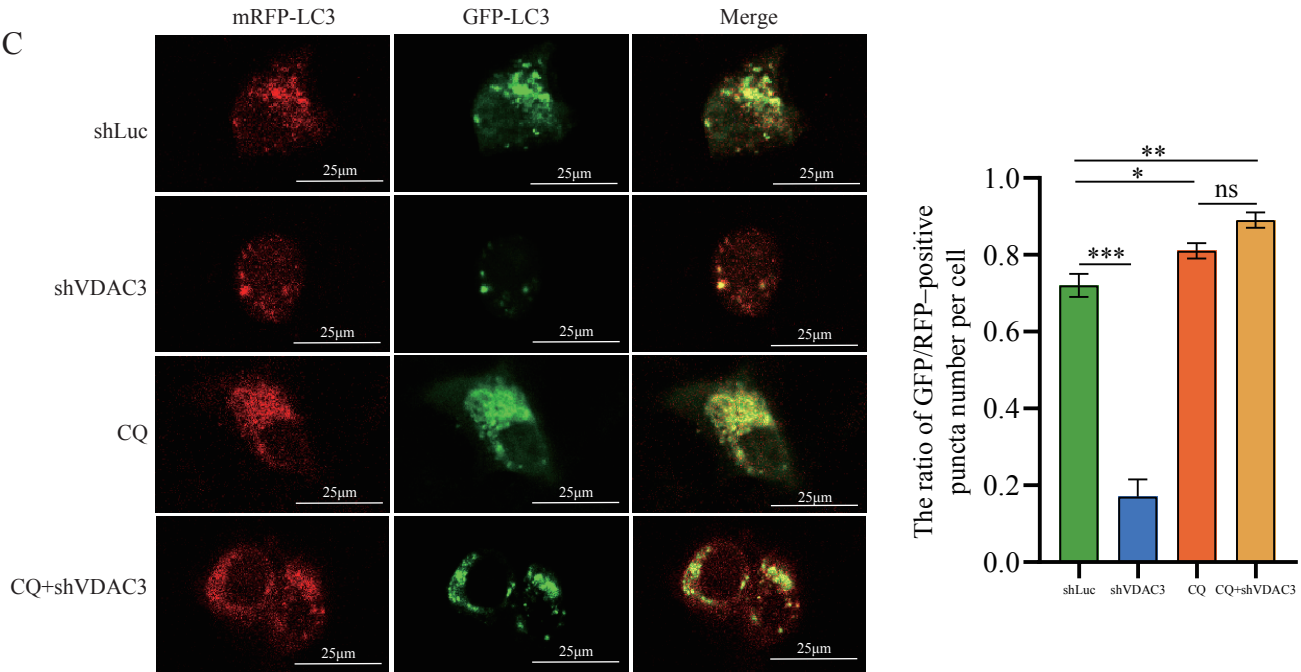

Supplement: Supplementary file 3 — Supplementary Figure 3 [file 41420_2026_2943_MOESM3_ESM.pdf]

A

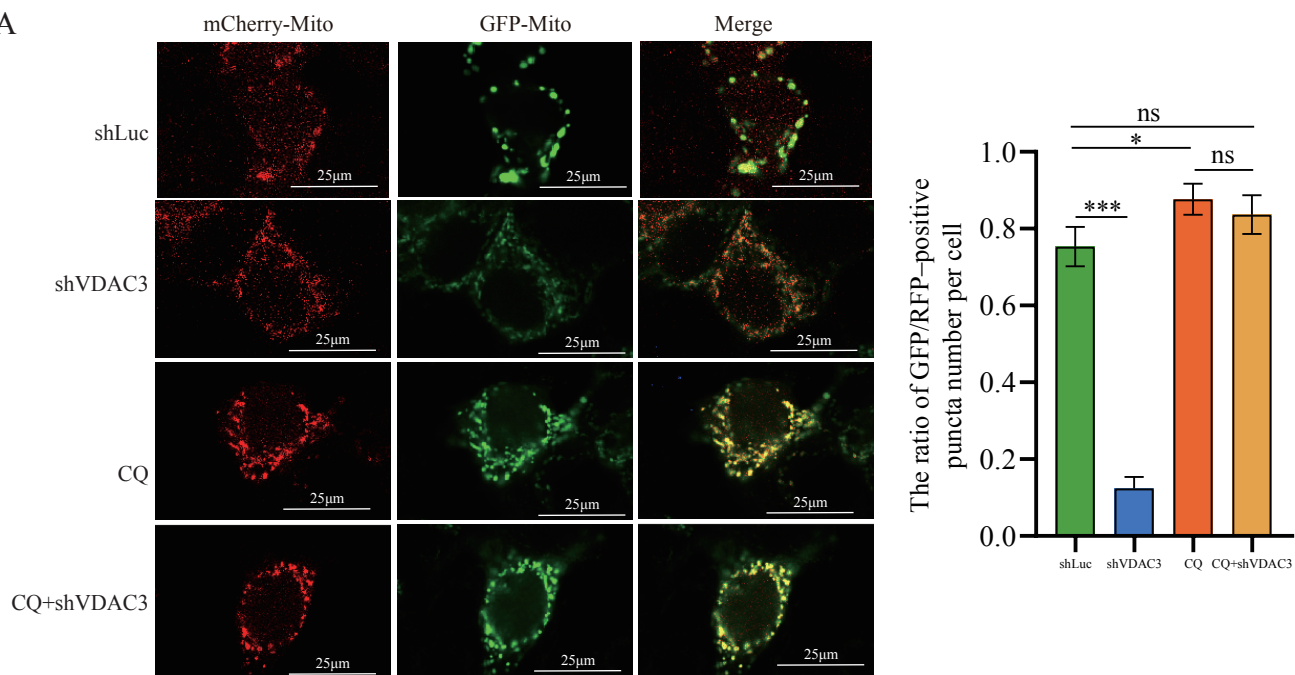

B

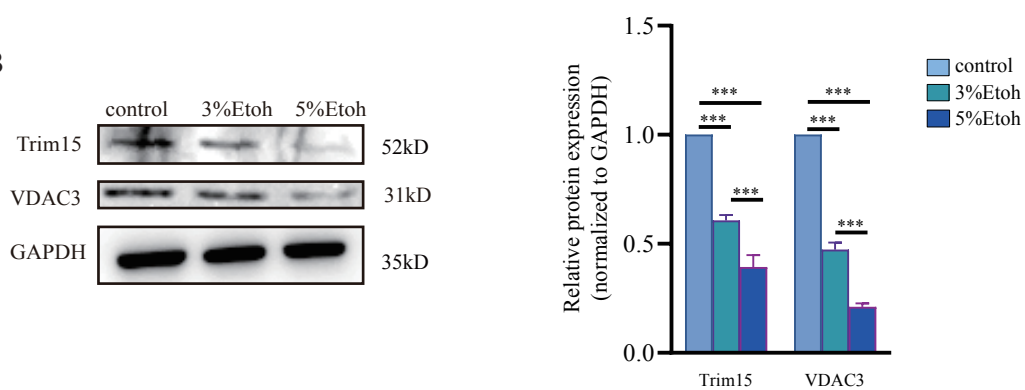

C

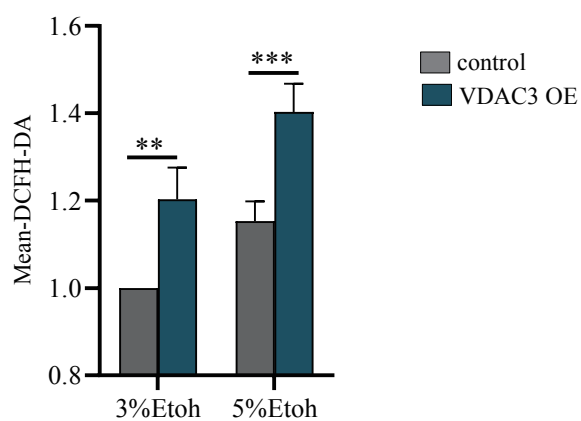

D

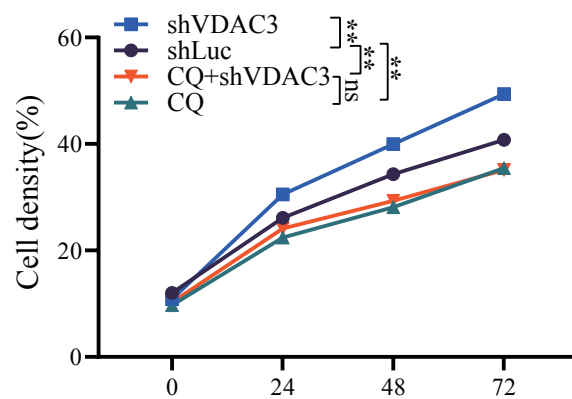

E

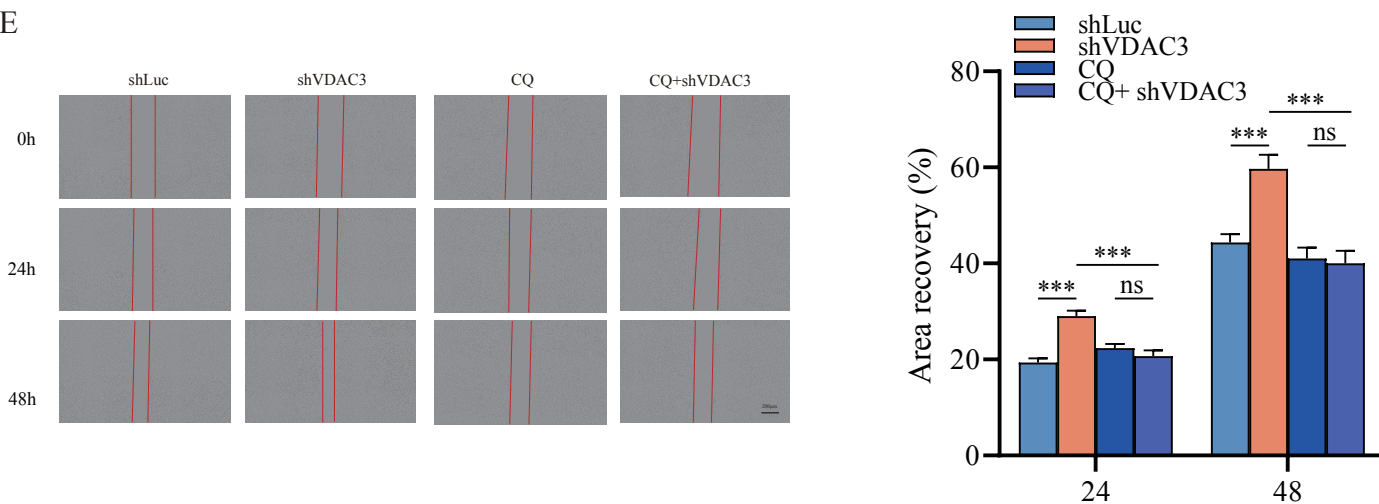

Supplement: Supplementary file 4 — Supplementary Figure 4 [file 41420_2026_2943_MOESM4_ESM.pdf]

A

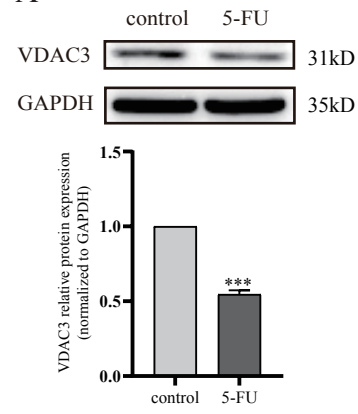

B

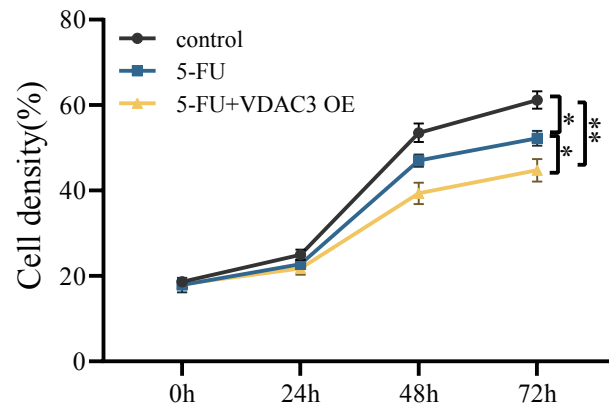

C

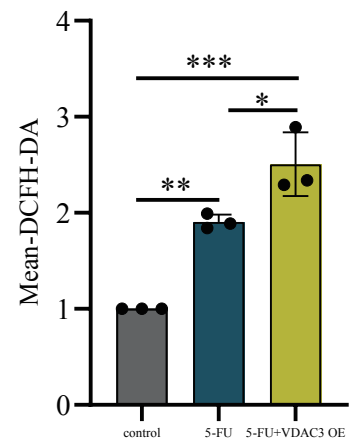

D

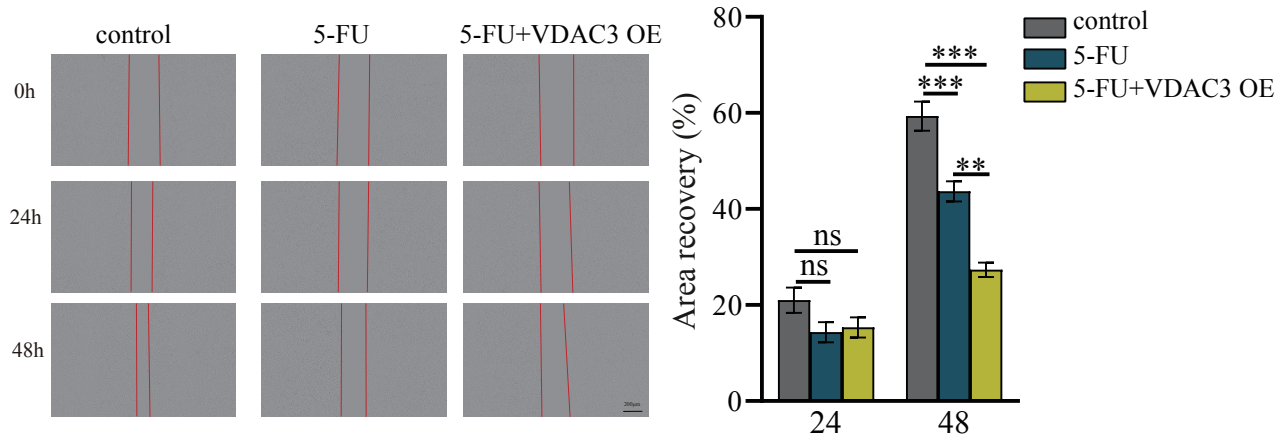

E

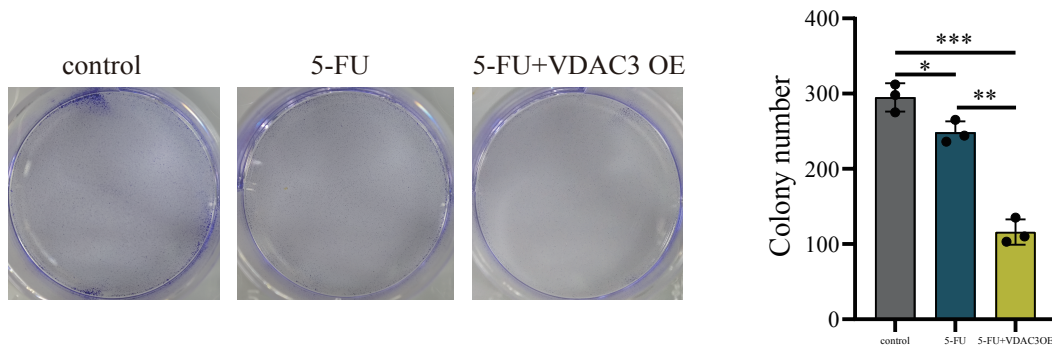

Supplement: Supplementary file 5 — Supplementary Figure 5 [file 41420_2026_2943_MOESM5_ESM.pdf]

B

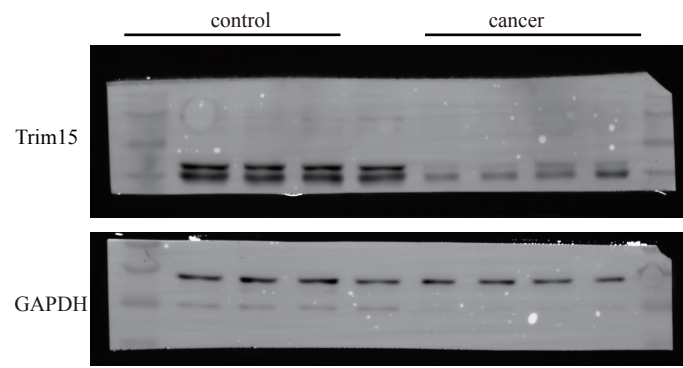

C

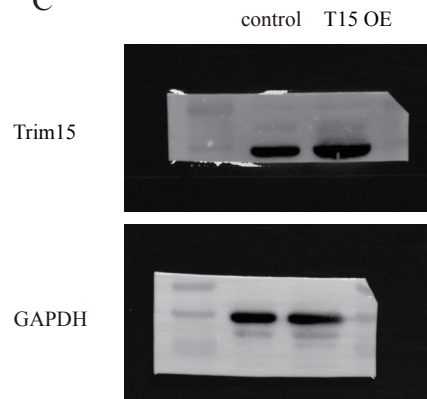

D

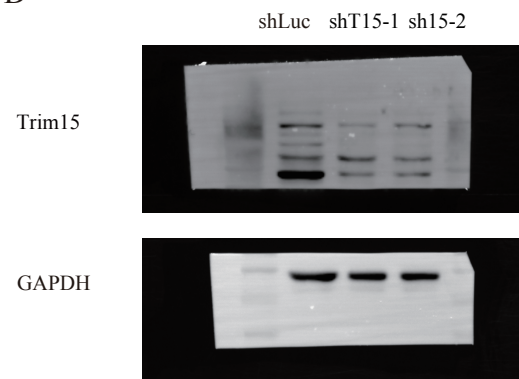

Supplement: Supplementary file 6 — Original images of Fig1 [file 41420_2026_2943_MOESM6_ESM.pdf]

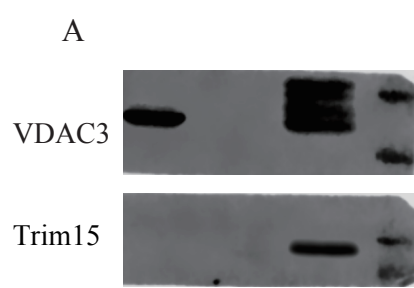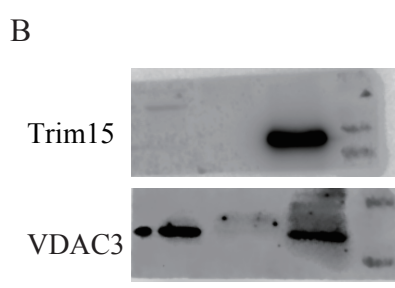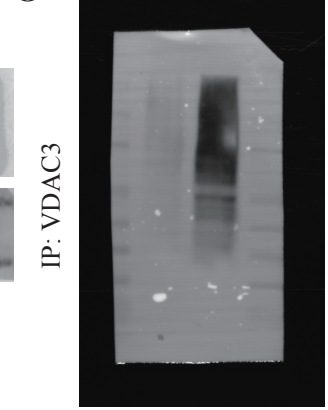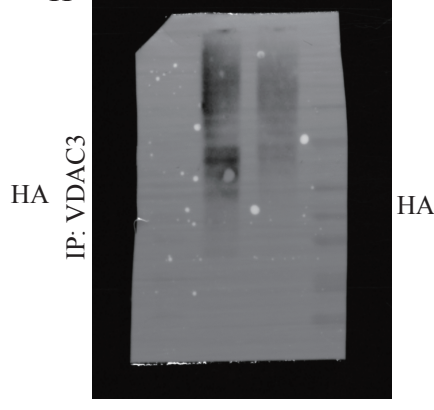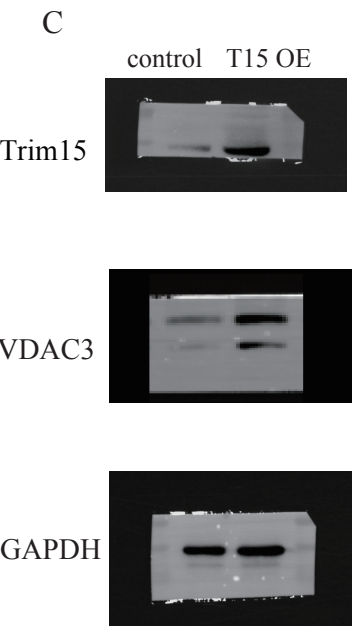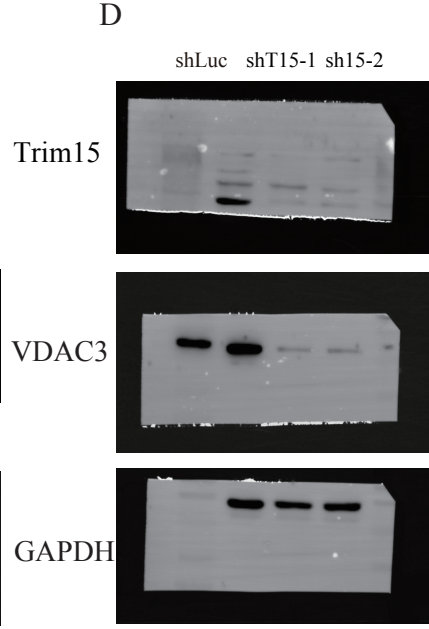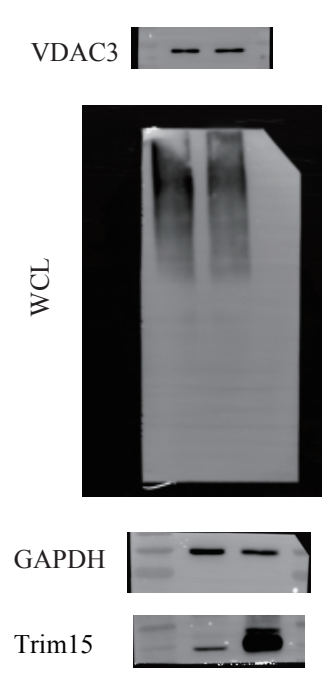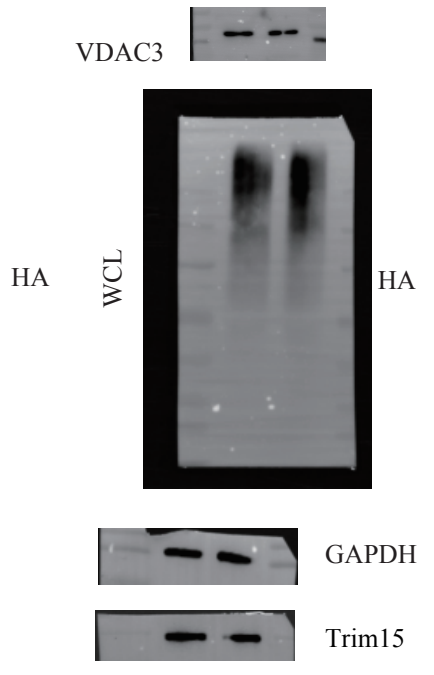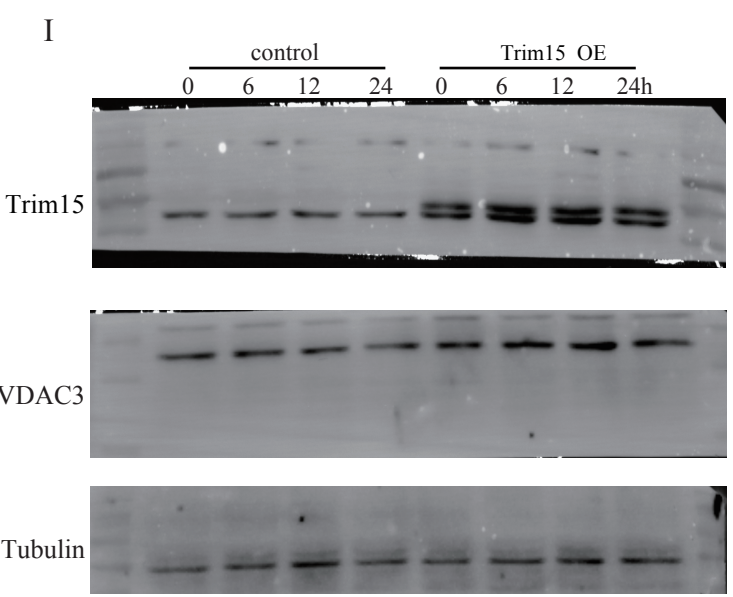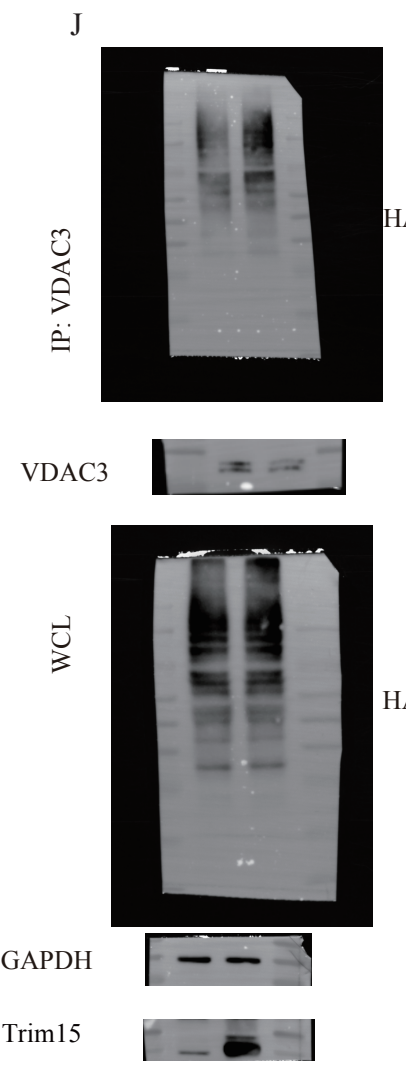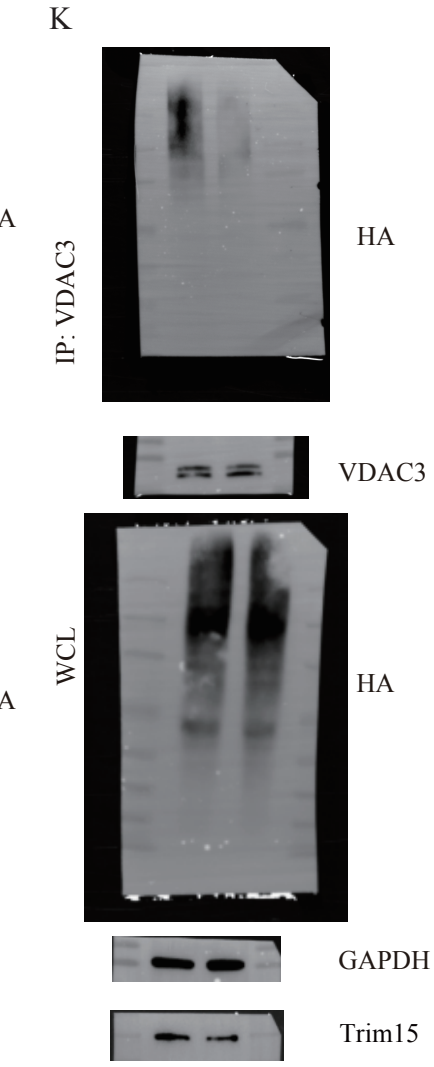

Supplement: Supplementary file 7 — Original images of Fig2 [file 41420_2026_2943_MOESM7_ESM.pdf]

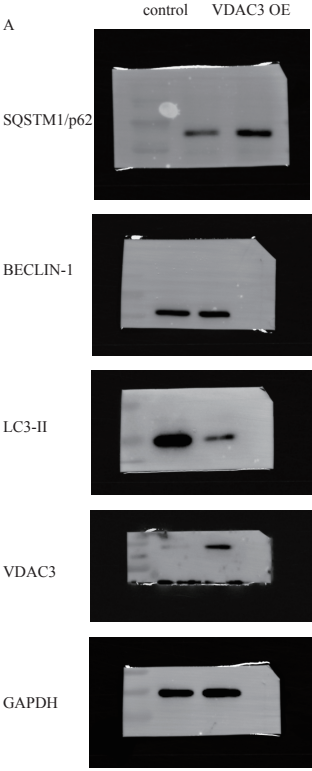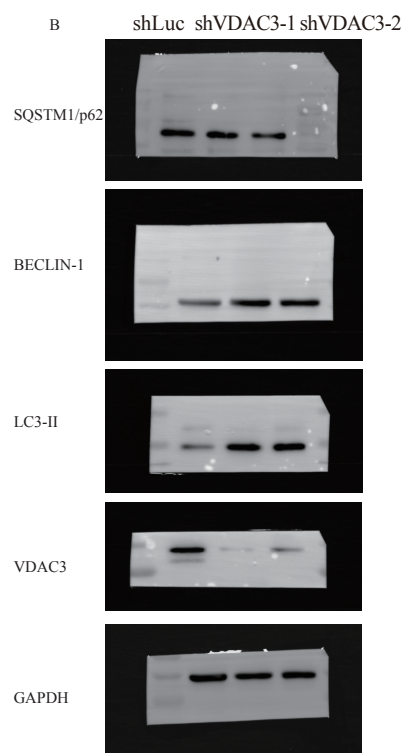

Supplement: Supplementary file 8 — Original images of Fig3 [file 41420_2026_2943_MOESM8_ESM.pdf]

A

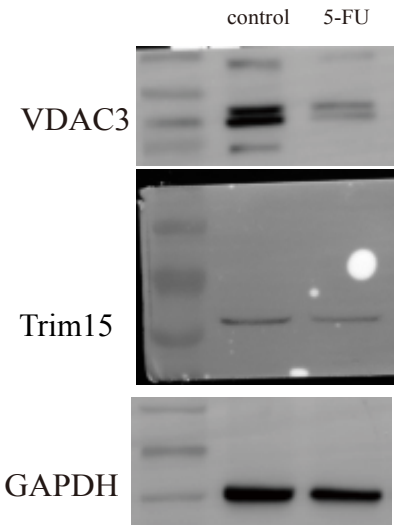

Supplement: Supplementary file 9 — Original images of Fig5 [file 41420_2026_2943_MOESM9_ESM.pdf]

F

control 3%Etoh 5%Etoh

Trim15

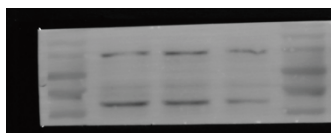

VDAC3

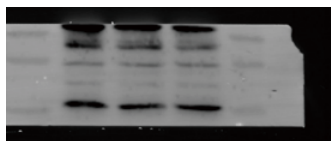

GAPDH

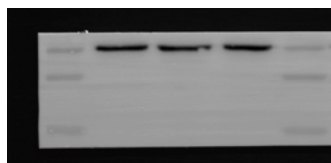

Supplement: Supplementary file 10 — Original images of Fig4 [file 41420_2026_2943_MOESM10_ESM.pdf]

E

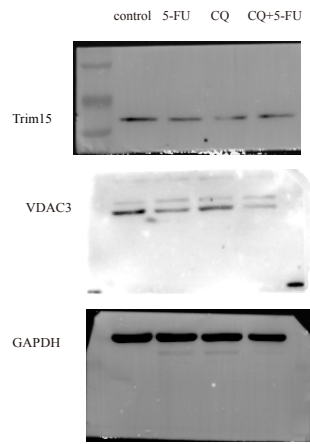

Supplement: Supplementary file 11 — Original images of Fig6 [file 41420_2026_2943_MOESM11_ESM.pdf]

A

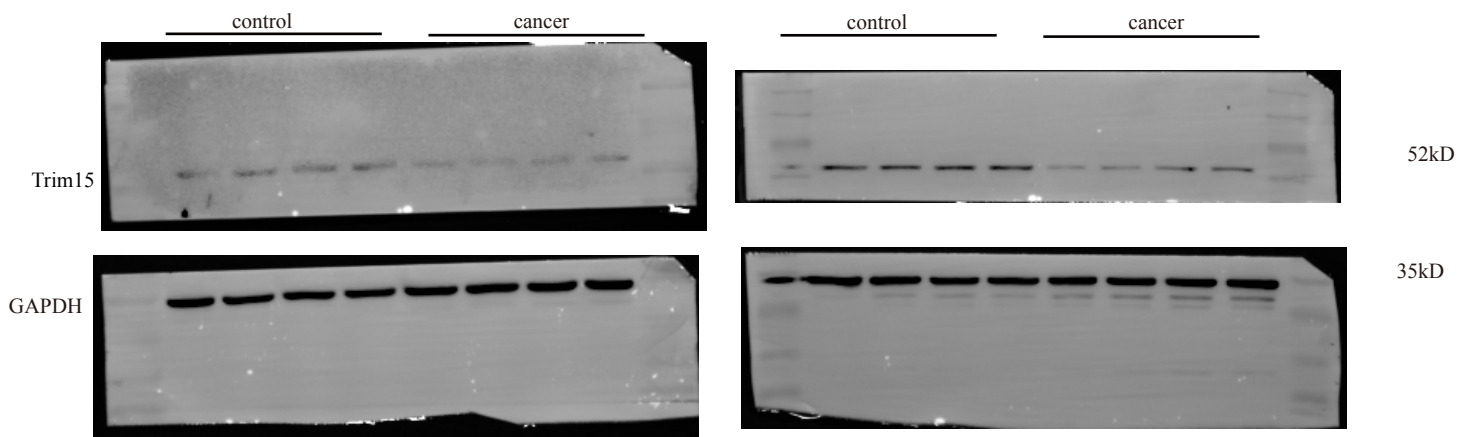

B

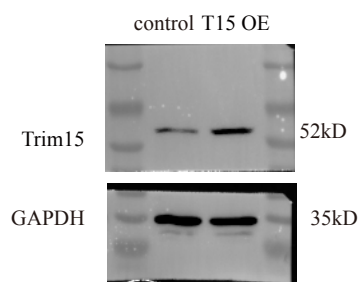

C

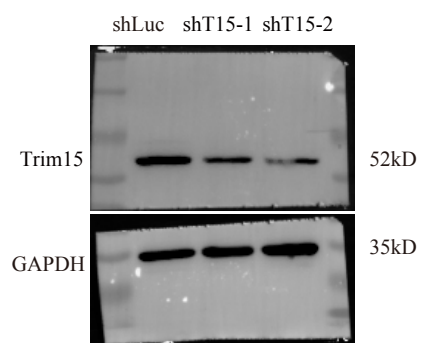

Supplement: Supplementary file 12 — Original Image of Supplementary Fig1 [file 41420_2026_2943_MOESM12_ESM.pdf]

B

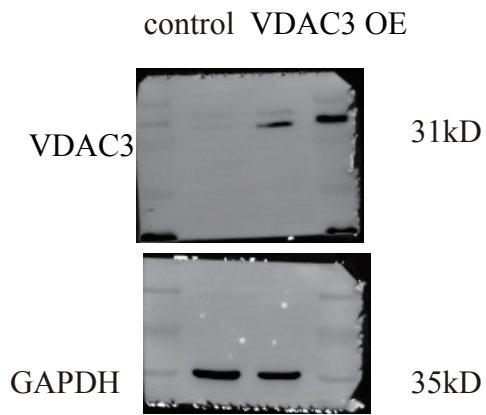

C

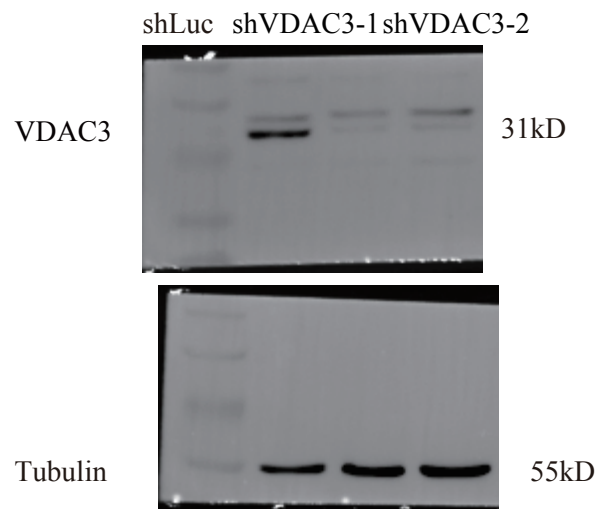

H

Ctrl + -  
TRIM15 - +  
HA-K48 + +

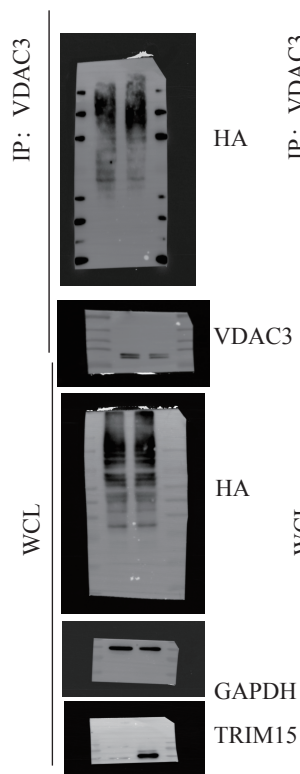

I

shLuc + -  
shTRIM15 - +  
HA-K48 + +

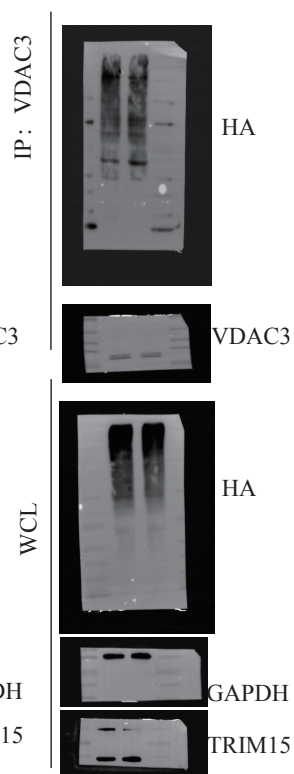

J

Ctrl + -  
TRIM15 - +  
HA-K48 + +

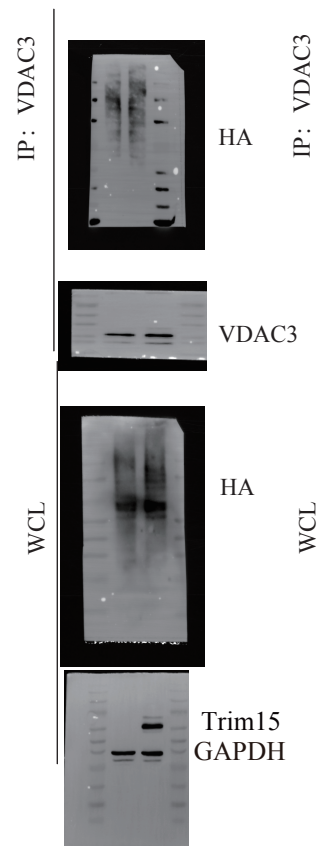

K

shLuc + -  
shTRIM15 - +  
HA-K48 + +

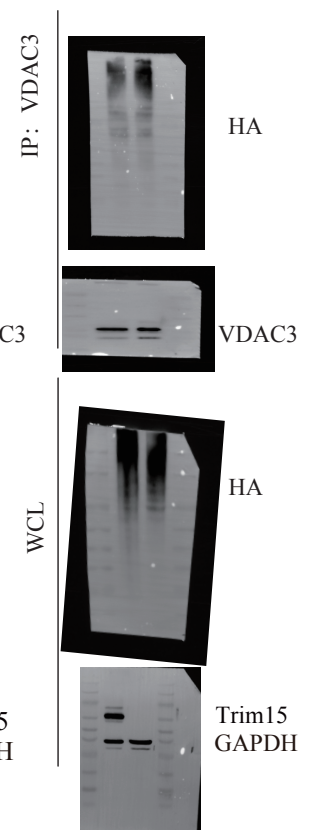

Supplement: Supplementary file 13 — Original Image of Supplementary Fig2 [file 41420_2026_2943_MOESM13_ESM.pdf]

A

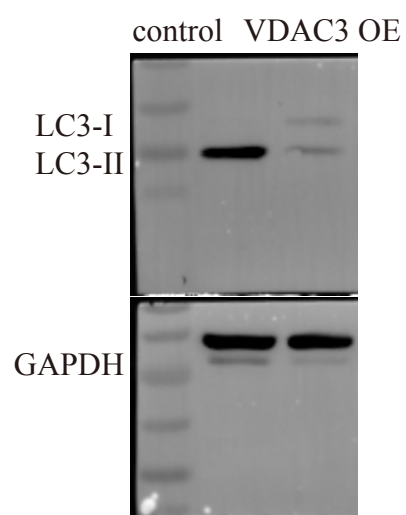

B

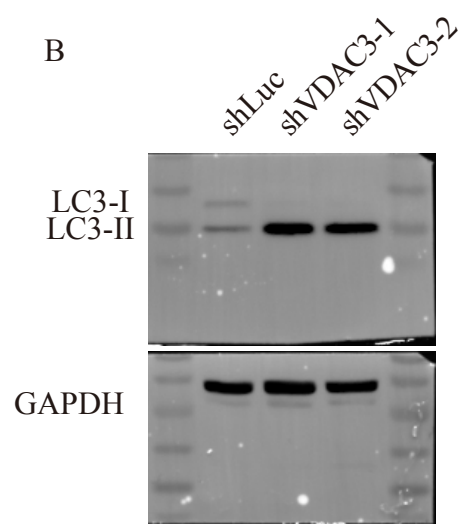

Supplement: Supplementary file 14 — Original Image of Supplementary Fig3 [file 41420_2026_2943_MOESM14_ESM.pdf]

B

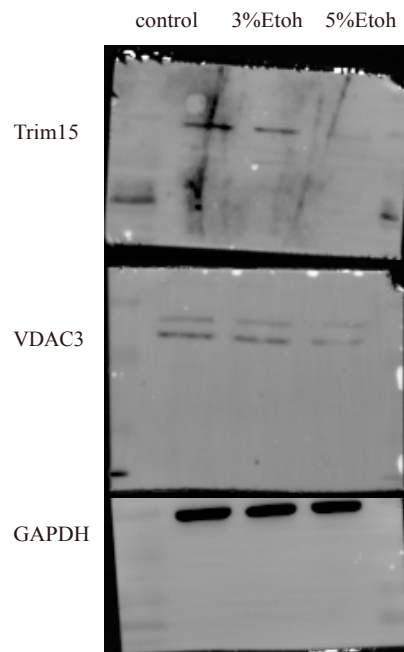

Supplement: Supplementary file 15 — Original Image of Supplementary Fig4 [file 41420_2026_2943_MOESM15_ESM.pdf]

A

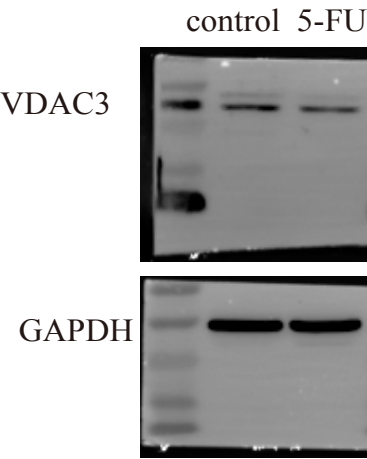

Supplement: Supplementary file 16 — Original Image of Supplementary Fig5 [file 41420_2026_2943_MOESM16_ESM.pdf]
