# Supplementary material for: Trim15 stabilizes VDAC3 via ubiquitination to suppress autophagy and enhance chemosensitivity in hypopharyngeal squamous cell carcinoma
Source: Cell Death Discov. 2026 Jan 30;12:88. doi: 10.1038/s41420-026-02943-0 (PMC12876940; doi:10.1038/s41420-026-02943-0)
Supplement: Supplementary file 17 — Supplementary Figure Legends [file 41420_2026_2943_MOESM17_ESM.pdf]

Supplementary Figure Legends

Supplementary Figure 1. Trim15 is downregulated in HPSCC and suppresses Detroit 562 cell proliferation and migration.

(A) Representative Western blot of Trim15 protein in the same tissue pairs.

(B, C) Western blot validation of Trim15 (B) overexpressionor (C) shRNA knockdown in Detroit 562 cells.

(D, E) Proliferation of Detroit 562 cells with Trim15 overexpression (D) or knockdown (E) versus controls, measured by IncuCyte live-cell imaging. Data are mean ± SEM (n=3 independent experiments); *P < 0.05, two-way ANOVA with Tukey's test.

(F, G) Wound-healing assays showing migration of Detroit 562 cells with Trim15 (F) overexpression (T15 OE) or (G) knockdown (shT15). Wound closure quantified at 24 and 48 hours. Data are mean ± SEM (n=3); **P < 0.01, ***P < 0.001, Student's t-test.

Supplementary Figure 2. VDAC3 suppresses Detroit 562 cell proliferation and migration.

(A) The coomassie blue-stained gel of Trim15 co-immunoprecipitation.

(B, C) Western blot validation of VDAC3 (B) overexpressionor (C) shRNA knockdown in Detroit 562 cells.

(D, E) Proliferation of Detroit 562 cells with VDAC3 overexpression (D) or knockdown (E) versus controls, measured by IncuCyte live-cell imaging. Data are mean ± SEM (n=3 independent experiments); ***P < 0.001, two-way ANOVA with Tukey's test.

(F, G) Wound-healing assays showing migration of Detroit 562 cells with VDAC3 (F) overexpression or (G) knockdown. Wound closure quantified at 24 and 48 hours. Data are mean ± SEM (n=3); n.s., not significant, *P < 0.05, ***P < 0.001, Student's t-test.

(H-K) Ubiquitination assays detecting K48- or K63-linked VDAC3 ubiquitination in FaDu cells with Trim15 overexpression or knockdown.

Supplementary Figure 3. VDAC3 regulates autophagy in Detroit 562 cells.

(A, B) Western blot and quantification analysis of autophagy markers LC3B in Detroit

562 cells with VDAC3 overexpression (A) or knockdown (B).

(C) mRFP-GFP-LC3 assay assessing autophagy flux in Detroit 562 cells. Images and autophagic puncta quantification shown. Data are mean ± SEM; *P < 0.05, **P < 0.01, ***P < 0.001, Student's t-test.

Supplementary Figure 4. VDAC3 modulates mitophagy, ROS, and cellular behavior in Detroit 562 cells.

(A) Cox8-GFP-mCherry assay showing mitophagic flux in Detroit 562 cells under control (shLuc), VDAC3 knockdown (shVDAC3), CQ, or combined conditions. Images and autophagic puncta quantification shown. Data are mean ± SEM; *P < 0.05, ***P < 0.001, n.s., not significant, Student's t-test.

(B) Western blot and quantification of Trim15 and VDAC3 in Detroit 562 cells post-alcohol (Etoh) treatment for 24 hours. Data are mean ± SEM; ***P < 0.001, Student's t-test.

(C) Flow cytometry analysis of ROS levels in Detroit 562 cells with VDAC3 overexpression in the presence of alcohol (Etoh). Data are mean ± SEM; **P < 0.01, ***P < 0.001, Student's t-test.

(D) Proliferation of Detroit 562 cells with control (shLuc), VDAC3 knockdown (shVDAC3), CQ, or combined treatment, measured by IncuCyte live-cell imaging. Data are mean ± SEM; **P < 0.01, n.s., not significant, two-way ANOVA with Tukey's test.

(E) Wound-healing assays showing migration of Detroit 562 cells in conditions as in (D) Wound closure quantified at 24 and 48 hours. Data are mean ± SEM; ***P < 0.001, n.s., not significant, Student's t-test.

Supplementary Figure 5. VDAC3 enhances 5-Fluorouracil (5-FU) sensitivity in Detroit 562 cells.

(A) Western blot and quantification of VDAC3 in Detroit 562 cells post-5-FU treatment.

(B) Proliferation assay in VDAC3-overexpressing Detroit 562 cells with 10 μM 5-FU. Data are mean ± SEM (n=3); *P < 0.05, **P < 0.01, two-way ANOVA with Tukey's test.

(C) Flow cytometry of ROS levels in conditions as in (B). Data are mean ± SEM (n=3); *P < 0.05, **P < 0.01, ***P < 0.001, Student's t-test.

(D) Wound-healing assay of VDAC3-overexpressing Detroit 562 cells with 10 μM 5-FU. Wound closure quantified at 24 and 48 hours. Data are mean ± SEM (n=3); ***P < 0.001, **P < 0.01, n.s., not significant, Student's t-test.

(E) Colony formation assay showing clonogenic survival in VDAC3-overexpressing Detroit 562 cells with 10 μM 5-FU. Data are mean ± SEM (n=3); *P < 0.05, **P < 0.01, ***P < 0.001, Student's t-test.
